# Structurally Various Sorbicillinoids From an Endophytic Fungus *Acremonium citrinum* SS-g13

**DOI:** 10.3389/fmicb.2022.800626

**Published:** 2022-03-23

**Authors:** Xiao-Ping Peng, Gang Li, Li-Mei Wang, Qi Wang, Cong Wang, Li-Xia Ji, Chen-Xi Cao, Guo-Feng Lin, Zu-Yang Jiang, Zhuo-qian He, Pei Wang, Hong-Xiang Lou

**Affiliations:** ^1^Department of Natural Medicinal Chemistry and Pharmacognosy, School of Pharmacy, Qingdao University, Qingdao, China; ^2^Guangxi Key Laboratory of Chemistry and Engineering of Forest Products, Guangxi University for Nationalities, Nanning, China; ^3^Hainan Key Laboratory of Research and Development of Natural Product From Li Folk Medicine, Hainan Institute for Tropical Agricultural Resources, Institute of Tropical Bioscience and Biotechnology, Chinese Academy of Tropical Agricultural Sciences, Haikou, China; ^4^Key Laboratory of Chemical Biology of Ministry of Education, Department of Natural Product Chemistry, School of Pharmaceutical Sciences, Shandong University, Jinan, China

**Keywords:** sorbicillinoid, trisorbicillinoid, *Acremonium*, endophyte, *Fructus mori*

## Abstract

Three new sorbicillinoids, including trimer trisorbicillinone E (**1**), acremosorbicillinoids A and B (**2** and **3**), and a new alkaloid acremokaloid A (**4**), and a new natural product 2*S*,3*S*-acetyl-β-methyltryptophan (**5**), were isolated from an endophytic fungus *Acremonium citrinum* SS-g13, which is found in *Fructus mori* plant root. In addition, eight known sorbicillinoids (**6–13**) were also obtained. The new compound structures were established using NMR, HRESIMS spectra, and reported spectroscopic data. The absolute configurations of compounds **1–5**, were determined by spectroscopic analysis, Snatzke’s method, and time-dependent density functional theory-electronic circular dichroism (TDDFT-ECD) calculations. Compound **11** exhibited significant cholesterol efflux enhancing activity. A plausible biosynthesis pathway for the sorbicillinoids is discussed.

## Graphical Abstract

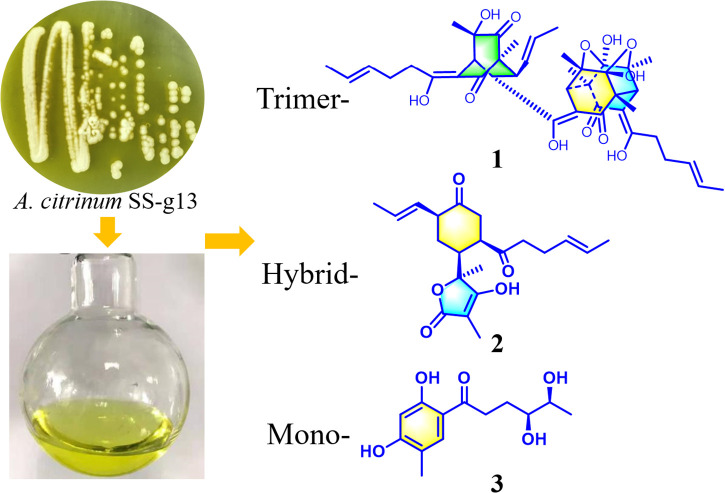

## Introduction

The sorbicillinoids family contains hexaketide metabolites with complex, highly oxygenated, bicyclic and tricyclic skeletons (Harned and Volp, 2011). Since the first report in 1948 (Cram, 1948; Cram and Tishler, 1948), there has been an increasing number of isolated sorbicillinoids, obtained from terrestrial and marine derived fungi. And these molecules were classified into the following groups according to the number of sorbicillinoid construction units: monomeric sorbicillinoids, bisorbicillinoids, trisorbicillinoids, and hybrid sorbicillinoids (Supplementary Figure 1; Meng et al., 2016). The carbon–carbon bond construction of sorbicillinoids in synthetic chemistry are Diels-Alder [4 + 2] (DA) and Michael [1 + 4] addition reactions. Due to their unique structures and diverse biological activities, sorbicillinoids have been subjects of interest in biosynthetic and synthetic studies in recent years (Meng et al., 2016; Kahlert et al., 2020; Chakrabarty et al., 2021).

**FIGURE 1:**
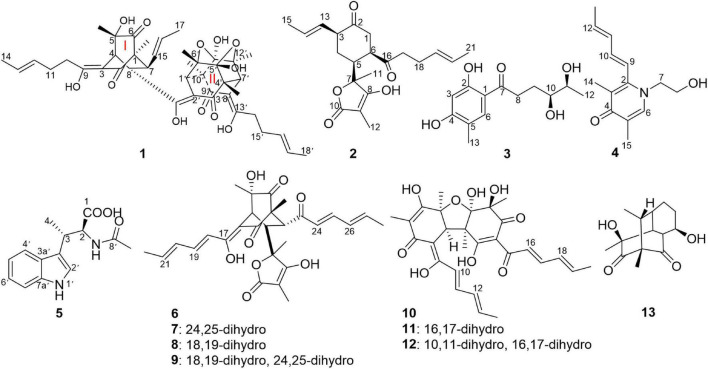
Structures of compounds **1–13**.

An investigation on the endophytic fungus *Acremonium citrinum* SS-g13 was previously carried out using 44 flasks containing rice culture media, resulting in three known sorbicillinoids (Peng et al., 2020). In our continuing search for novel sorbicillinoid-type secondary metabolites from this fungus, a large-scale culture strategy (Hu et al., 2021) was applied to explore its chemical diversity. In this study, 80 flasks were used to ferment this fungal strain. The fungal crude extract chemical investigation led to the isolation of three new sorbicillinoids, including trisorbicillinone E (**1**) and acremosorbicillinoids A (**2**) and B (**3**), one new alkaloid acremokaloid A (**4**), and the new natural product 2*S*,3*S*-acetyl-β-methyltryptophan (**5**). Among the products, trisorbicillinone E (**1**) was a novel trimeric sorbicillinoid. To the best of our knowledge, this kind of trimeric compound is rare in nature. Furthermore, only six trisorbicillinoids (Supplementary Figure 1; Li et al., 2007, 2010; Guo et al., 2013; Cao et al., 2020) have been discovered in nature, mainly from marine fungi. The other two sorbicillinoids, acremosorbicillinoids A and B (**2** and **3**), were hybrid and monomeric sorbicillinoids, respectively. In addition to the five new compounds isolated from the fungus *A. citrinum* SS-g13, seven known bisorbicillinoids, i.e., trichotetronine (**6**) (Shirota et al., 1997), dihydrotrichotetronine (**7**) (Shirota et al., 1997), 10,11-dihydrobislongiquinolide (**8**) (Yu et al., 2019), 10,11,16,17-tetrahydrobislongiquinolide (**9**) (Yu et al., 2019), bisvertinolone (**10**) (Andrade et al., 1992), dihydrobisvertinolone (**11**) (Liu et al., 2005b), tetrahydrobisvertinolone (**12**) (Liu et al., 2005b), and one known mono-sorbicillinoid penicillone B (**13**) (Liu et al., 2005c), were also discovered (Figure 1). The planar and spatial structures of these sorbicillinoids were determined. This study describes the isolation, structural elucidation, and biological activities of these isolates.

## Materials and Methods

### General Experimental Procedures

Optical rotations were measured on a Perkin-Elmer 241MC polarimeter (Perkin-Elmer Instruments, Norwalk, CT, United States) in MeOH at 20°C. Electronic circular dichroism (ECD) spectra were acquired on a Chirascan spectropolarimeter (Applied Photophysics, United Kingdom). Infrared (IR) spectra were recorded on a Nicolet iN10 (Thermo Fisher Scientific, Waltham, MA, United States). Nuclear magnetic resonance (NMR) spectra were obtained on JNM-ECP 600 (JEOL, Japan), DD2-500 (Agilent, United States), and AVANCE NEO 400 (Bruker, United States) operating at 600/500/400 (^1^H) and 150/125 (^13^C) MHz, using DMSO-*d*_6_ and CDCl_3_ as the solvent, with tetramethylsilane (TMS) as an internal standard. Low-resolution mass spectra (Applied Biosystems, United States) were obtained on a LTQ Orbitrap spectrometer equipped with an electrospray ionization (ESI) source. High-resolution electrospray ionization mass spectrometry (HRESIMS) data were determined on a Finnigan LC-QDECA mass spectrometer (Thermo Electron, San Jose, Calif., United States). The semi-preparative HPLC system (Agilent 1260 Infinity II, Agilent Technologies, Germany) was equipped with a 1260 Quat Pump VL, a 1260 Vialsampler, a 1260 multicolumn thermostat (MCT), a 1260 diode array detector (DAD) WR, and a ZORBAX SB-C18 column (5 μm, 9.4 × 250 mm).

### Fungal Material

The endophytic fungus *A. citrinum* SS-g13 was isolated from the root of the terrestrial plant *F. mori*, collected in February 2017 from Dezhou, Shandong, People’s Republic of China. The fungus was identified according to its morphological characteristics and the analysis of the rDNA internal transcribed spacer (ITS) region (GenBank access number MK034752). This fungus was stored at −80°C at the School of Pharmacy, Qingdao University, People’s Republic of China.

### Extraction and Isolation

The *A. citrinum* SS-g13 fungus was cultured on plates with potato dextrose agar medium at 28°C for 4 days. The fungal strain agar plugs were inoculated into 80 flasks (500 mL), each containing 80 g of rice, 0.24 g of peptone, and 120 mL of tap-water. The flasks were incubated under static conditions at 28°C. After 70 days of cultivation, the fermented cultures were extracted 3 times with ethyl acetate (EtOAc, 16 L × 3). The organic solvent was evaporated under reduced pressure to afford the crude extract (65.3 g).

Based on thin-layer chromatography (TLC) analysis, the crude extract was fractionated into six fractions (Fr.1–Fr.6) by column chromatography on silica gel, eluting with a gradient of CH_2_Cl_2_-MeOH (100–50%). Fr.2 (14.8 g) was fractionated by silica gel column chromatography with a gradient of EtOAc-petroleum ether (5–100%) to give eight subfractions (Fr.2.1–Fr.2.8). Fr.2.5 (2.9 g) was separated by a CombiFlash Rf 200 purification system (C18 spherical 20–35 μm 100A 80 g, 30 mL/min, eluting with a gradient of MeOH-H_2_O, from 60% MeOH to 80% MeOH over 50 min), obtaining six subfractions (Fr.2.5.1–Fr.2.5.6). Fr.2.5.6 (280.2 mg) was purified by semi-preparative HPLC (MeCN/H_2_O, 70:30, 2 mL/min, plus 0.1% FA) to obtain **1** (2.8 mg, *t*_R_ 21.5 min), **10** (32.7 mg, *t*_R_ 15.0 min), **11** (10.0 mg, *t*_R_ 16.5 min), and **12** (7.5 mg, *t*_R_ 18.0 min). Fr.2.6 (614.4 mg) was separated by a CombiFlash Rf 200 purification system (C18 spherical 20–35 μm 100A 80 g, 30 mL/min, eluting with MeOH-H_2_O, 75% MeOH for 25 min, and 100% MeOH for 10 min), affording four subfractions (Fr.2.6.1–Fr.2.6.4). Fr.2.6.1 (143.1 mg) was to obtain needle **13** (55.0 mg). Fr.2.6.2 (153.7 mg) was sequentially purified through semi-preparative HPLC (MeOH/H_2_O, 75:25, 2 mL/min, plus 0.1% FA) to give **9** (15.1 mg, *t*_R_ 21.7 min). Fr.2.7 (851.5 mg) was purified by semi-preparative HPLC (MeOH/H_2_O, 75:25, 2 mL/min, plus 0.1% FA) to obtain six subfractions (Fr.2.7.1–Fr.2.7.6). Fr.2.7.1 (48.7 mg) was purified using semi-preparative HPLC (MeCN/H_2_O, 45:55, 2 mL/min, plus 0.1% FA) to yield **2** (1.3 mg, *t*_R_ 27.0 min), and **6** (2.2 mg, *t*_R_ 43.0 min). Fr.2.7.3 (173.6 mg) was purified by semi-preparative HPLC (MeCN/H_2_O, 48:52, 2 mL/min, plus 0.1% FA) to yield **8** (10.7 mg, *t*_R_ 38.5 min). Fr.2.7.6 (92.1 mg) was separated by semi-preparative HPLC (MeCN/H_2_O, 50:50, 2 mL/min, plus 0.1% FA) to obtain **7** (15.6 mg, *t*_R_ 39 min). Similarly, Fr.5 (2.2 g) was applied to a CombiFlash Rf 200 purification system (C18 spherical 20–35 μm 100A 80 g, 30 mL/min, eluting with MeOH-H_2_O, 5% MeOH for 5 min, a gradient of 5% MeOH to 100% MeOH over 40 min), which obtained three subfractions (Fr.5.1–Fr.5.3). Fr.5.3 (71.5 mg) was subsequently purified by semi-preparative HPLC (MeOH/H_2_O, 42:58, 2 mL/min, plus 0.1% FA) to afford **3** (4.6 mg, *t*_R_ 21.0 min), **4** (5.3 mg, *t*_R_ 14.4 min), and **5** (7.3 mg, *t*_R_ 22.0 min), respectively.

Trisorbicillinone E (**1**): yellow oil; [α]^20^_D_ + 151.1 (*c* 0.080, MeOH); UV (MeOH) λ_max_ 202, 296 nm; IR ν_max_ 3,500, 2,922, 2,856, 1,732, 1,593, 1,448, 1,376, 1,264, 1,135, 1,007, 970 cm^–1^; ^1^H and ^13^C NMR data (see Table 1); HRESIMS *m/z* 749.3521 [M + H]^+^ (calcd. for C_42_H_53_O_12_ 749.3532).

**TABLE 1:** 1H NMR (500 MHz) and ^13^C NMR (125 MHz) data for 1 in CDCl_3_ (δ in ppm, J in Hz).

Position	δ_C_, type	δ_H_, (*J* in Hz)
1	62.3, C	
2	195.7, C	
3	107.8, C	
4	45.6, CH	3.04, s
5	75.8, C	
6	211.4, C	
7	50.6, CH	2.75, m
8	41.9, CH	3.61, d (6.9)
9	181.8, C	
10	31.9, CH_2_	2.16, m
11	28.5, CH_2_	2.28, m
12	129.3, CH	5.41, m
13	126.8, CH	5.50, m
14	18.0, CH_3_	1.66^a^, d (9.2)
15	127.8, CH	4.98, dd (14.6, 9.6)
16	130.4, CH	5.35, m
17	17.7, CH_3_	1.59, d (6.3)
18	190.6, C	
1′	57.8, CH	2.92, s
2′	103.7, C	
3′	195.1, C	
4′	57.9, C	
5′	103.9, C	
6′	78.7, C	
7′	57.9, CH	3.00, s
8′	104.3, C	
9′	193.1, C	
10′	57.6, C	
11′	104.0, C	
12′	78.8, C	
13′	192.5, C	
14′	34.7, CH_2_	2.43, m
15′	28.5, CH_2_	2.28, m
16′	129.1, CH	5.41, m
17′	126.7, CH	5.49, m
18′	18.0, CH_3_	1.64^a^, d (9.1)
CH_3_-1	9.9, CH_3_	1.11, s
CH_3_-5	24.9, CH_3_	1.26, s
CH_3_-4′	19.0, CH_3_	1.42, s
CH_3_-6′	21.1, CH_3_	1.30, s
CH_3_-10′	18.2, CH_3_	1.52, s
CH_3_-12′	21.3, CH_3_	1.44, s

*^a^The assignments could be interchanged.*

Acremosorbicillinoid A (**2**): yellow oil; [α]^20^_D_ + 20.0 (*c* 0.020, MeOH); UV (MeOH) λ_max_ 232 nm; IR ν_max_ 3,495, 2,928, 2,856, 1,725, 1,593, 1,435, 1,372, 1,231, 1,056, 816 cm^–1^; ^1^H and ^13^C NMR data (see Table 2); HRESIMS *m/z* 383.1833 [M + Na]^+^ (calcd. for C_21_H_28_O_5_Na 383.1829).

**TABLE 2:** ^1^H NMR (500 MHz) and ^13^C NMR (125 MHz) data for 2 in DMSO-*d*_6_ (δ in ppm, *J* in Hz).

Position	δ_C_, type	δ_H_, (*J* in Hz)
1	37.9, CH_2_	2.38, m; 2.33, m
2	210.5, C	
3	37.7, CH	2.82, dt (6.9, 11.3)
4	41.0, CH_2_	2.35, m; 2.05, m
5	48.7, CH	2.92, t (4.9)
6	40.0, CH	2.25, m
7	83.3, C	
8	131.6, C	
9	81.0, C	
10	177.6, C	
11	22.0, CH_3_	1.06, s
12	6.8, CH_3_	1.34, s
13	131.6, CH	5.22, dd (15.4, 7.3)
14	125.3, CH	5.31, m
15	17.7, CH_3_	1.56, d (6.8)
16	212.8, C	
17	43.6, CH_2_	2.27, m
18	25.8, CH_2_	2.04, m
19	130.3, CH	5.35, m
20	124.7, CH	5.35, m
21	17.6, CH_3_	1.58, d (6.5)

Acremosorbicillinoid B (**3**): yellow oil; [α]^20^_D_ + 7.0 (*c* 0.033, MeOH); UV (MeOH) λ_max_ 214, 234, 278, 326 nm; IR ν_max_ 3,495, 2,928, 1,724, 1,594, 1,425, 1,380, 1,230, 1,051, 822 cm^–1^; ^1^H and ^13^C NMR data (see Table 3); HRESIMS *m/z* 277.1053 [M + Na]^+^ (calcd. for C_13_H_18_O_5_Na 277.1046).

**TABLE 3:** ^1^H NMR (600 MHz) and ^13^C NMR (150 MHz) data for 3 in DMSO-*d*_6_ (δ in ppm, *J* in Hz).

Position	δ_C_, type	δ_H_, (*J* in Hz)
1	111.5, C	
2	164.0, C	
3	101.9, CH	6.29, s
4	162.8, C	
5	116.6, C	
6	132.2, CH	7.61, s
7	204.9, C	
8	34.0, CH_2_	3.03, m; 2.94, m
9	28.2, CH_2_	1.89, m; 1.53, m
10	74.2, CH	3.21, m ddd (2.9, 8.9, 6.2)
11	69.6, CH	3.40, dq (6.2, 6.2)
12	19.5, CH_3_	1.06, d (6.2)
13	15.3, CH_3_	2.05, s

Acremokaloid A (**4**): yellow oil; [α]^20^_D_ −0.9 (*c* 0.033, MeOH); UV (MeOH) λ_max_ 200, 246, 280 nm; IR ν_max_ 3,495, 1,632, 1,534, 1,497, 1,375, 1,285, 1,240, 1,076, 1,010, 682 cm^–1^; ^1^H and ^13^C NMR data (see Table 4); HRESIMS *m/z* 234.1485 [M + H]^+^ (calcd. for C_14_H_20_NO_2_ 234.1494).

**TABLE 4:** ^1^H NMR (500 MHz) and ^13^C NMR (125 MHz) data for 4 in DMSO-*d*_6_ (δ in ppm, *J* in Hz).

Position	δ_C_, type	δ_H_, (*J* in Hz)
2	144.4, C	
3	121.2, C	
4	176.7, C	
5	121.2, C	
6	138.5, CH	7.56, s
7	55.0, CH_2_	3.88, t (5.5)
8	60.1, CH_2_	3.56, m
9	121.3, CH	6.36, d (15.9)
10	138.8, CH	6.40, m
11	130.9, CH	6.29, dd (15.3, 7.4)
12	133.2, CH	5.94, m
13	18.1, CH_3_	1.80, d (6.7)
14	13.3, CH_3_	1.89, s
15	13.8, CH_3_	1.84, s
8-OH		4.96, br s

2*S*,3*S*-Acetyl-β-methyltryptophan (**5**): yellow oil; [α]^20^_D_ + 33.6 (*c* 0.033, MeOH); UV (MeOH) λ_max_ 196, 248, 290 nm; ^1^H and ^13^C NMR data (see Table 5); HRESIMS *m/z* 261.1234 [M + H]^+^ (calcd. for C_14_H_17_N_2_O_3_ 261.1234).

**TABLE 5:** ^1^H NMR (600 MHz) and ^13^C NMR (150 MHz) data for 5 in DMSO-*d*_6_ (δ in ppm, *J* in Hz).

Position	δ_C_, type	δ_H_, (*J* in Hz)
1	173.6, C	
2	57.2, CH	4.57, dd (8.6, 6.0)
3	32.3, CH	3.57, m
4	16.6, CH_3_	1.31, d (7.1)
1′		10.80, s
2′	122.2, CH	7.12, s
3′	116.4, C	
3a′	126.5, C	
4′	118.4, CH	7.54, d (7.9)
5′	118.1, CH	6.97, t (7.4)
6′	120.7, CH	7.05, t (7.5)
7′	111.4, CH	7.32, d (8.1)
7a′	136.1, C	
8′	169.2, C	
9′	22.5, CH_3_	1.83, s
NH		7.94, d (8.8)

Crystallographic data for penicillone B (**13**). Molecular formula, C_14_H_20_O_4_ (*M* = 252.30 g/mol), orthorhombic, space group = P2_1_2_1_2_1_; unit cell dimensions: a = 8.4852(4) Å, b = 10.2957(5) Å, c = 14.7986(8) Å, V = 1292.82(11) Å^3^, ρ_calcd_ = 1.296 g/cm^3^, Z = 4, T = 296.00(2) K, μ(CuKα) = 0.770 mm^–1^, A total of 9,554 reflections were measured (10.466° ≤ 2Θ ≤ 133.170°) with 2,266 independent reflections (R_int_ = 0.0539, R_sigma_ = 0.0457). Final R indexes [I > = 2σ (I)]: R_1_ = 0.0453, wR_2_ = 0.1092. Final R indexes [all data]: R_1_ = 0.0510, wR_2_ = 0.1130. Largest diff. peak and hole = 0.225 and −0.275 eÅ^–3^. Flack parameter = −0.02 (15). Crystallographic data (excluding structure factors) for compound **13** in this paper have been deposited with the Cambridge Crystallographic Data Centre as supplementary publication number CCDC 2,081,490.

### Biological Assays

The cytotoxicity of the five new compounds (**1**-**5**) was tested using the MTT assay (Xu et al., 2018) against the human cancer cell lines (A2870, HepG2, EC109, PC3, and A549) and the human bronchial epithelial cell line. Compounds **1**-**5** were also evaluated for the antibacterial activities against Gram-positive bacteria *Staphylococcus aureus* and *Bacillus subtilis*, and Gram-negative bacteria *Pseudomonas aeruginosa* and *Escherichia coli* by using the disk diffusion method (Pfaller and Jones, 2006). All the compounds (**1**-**13**) were tested for their quorum sensing (QS) inhibitory activity against *Chromobacterium violaceum* CV026, using a previously described method (Zhang et al., 2019). All the compounds were also tested against agricultural pathogenic fungi *Colletotrichum musae* (ACCC 31244), *C. coccodes* (ACCC 36067), *Fusarium solanum*, *F. oxysporum f.* sp. *cubense*, *Cucumber fusarium wilt*, *Cowpea wilt*, *F. graminearum*, *Nectria* sp., *F. mangiferae*, *C. asianum, and Alternaria solani*., using a disk diffusion and half dilution method (Zhang et al., 2019). Effect of know compounds **6**, **7**, **8**, **11**, and **12** on serum-mediated cholesterol efflux and on cell viability in J774A.1 macrophages were also tested (Sankaranarayanan et al., 2011; Wang et al., 2017; Hou et al., 2021).

## Results and Discussion

### Structural Elucidation of the Isolated Compounds

**FIGURE 2:**
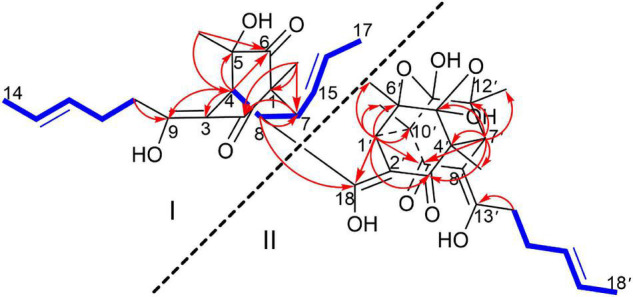
^1^H-^1^H COSY (blue bold lines), Key HMBC (red arrows) correlations of trisorbicillinone E (**1**).

Trisorbicillinone E (**1**) was isolated as a yellow oil. The infrared (IR) spectrum of **1** (Supplementary Figure 11) indicated that hydroxy and carbonyl signals were present at 3,500 and 1,731 cm^–1^, respectively. The molecular formula of **1**, C_42_H_52_O_12_ with seventeen unsaturation degrees, was established from a positive HRESIMS ion at 749.3521 [M + H]^+^ (calcd. for 749.3532 C_42_H_53_O_12_) (Supplementary Figure 10). Analysis of the 1D NMR spectroscopic data (Table 1) with the aid of the HSQC spectrum determined that **1** possessed six sp^3^ methyls (δ_C_ 9.9, 24.9, 19.0, 21.1, 18.2, and 21.3), three methyl groups attached to double bonds (δ_C_ 18.0, 17.7, and 18.0), five sp^3^ methines (δ_C_ 45.6, 50.6, 41.9, 57.8, and 57.9), and three 1,2-disubstituted double bonds (δ_C_ 129.3 and 126.8, 127.8 and 130.4, and 129.1 and 126.7). These structural features were similar to those of trisorbicillinone D (Li et al., 2010; Supplementary Figure 43), implying that **1** was also a sorbicillin trimer. By carefully comparing the 1D NMR data of compound **1** and trisorbicillinone D, two major differences were found. The first one was the absence of four olefinic carbon atoms in **1**. This distinct feature indicated that in compound **1**, two of the sorbyl side chains from C-9 to C-14 and from C-13′ to C-18′ might be partially hydrogenated. Another difference was the presence of a methyl group (17-CH_3_) at 1.59 ppm (H-17, 3H, d, *J* = 6.3 Hz) in **1** in contrast with the methyl group at 0.92 ppm (3H, d, *J* = 7.2 Hz) in trisorbicillinone D. Additionally, the ^1^H-^1^H COSY correlations (Figure 2) of H-4/H-8/H-7/H-15/H-16/3H-17 secured the presence of a prop-1-en-1-yl substituent at C-7 in **1**. The different chemical shifts on the methyl mentioned above and the COSY and HMBC correlations (Supplementary Figure 44) suggested the structural changes proposed in compound **1** with respect to trisorbicillinone D.

To unambiguously deduce the planar structure of **1**, the HMBC spectrum was analyzed (Figure 2). In fragment I, the key HMBC correlations from CH_3_-5 (δ_H_ 1.26) to C-6 (δ_C_ 211.4) and C-4 (δ_C_ 45.6), from CH_3_-1 (δ_H_ 1.11) to C-1 (δ_C_ 62.3), C-2 (δ_C_ 195.7) and C-7 (δ_C_ 50.6), from H-7 (δ_H_ 2.75) to C-2 (δ_C_ 195.7), and from H-4 (δ_H_ 3.04) to C-3 (δ_C_ 107.8), C-5 (δ_C_ 75.8), C-6 and C-9 (δ_C_ 181.8) were assigned to an [2,2,2] octane group monomer. In addition, the ^1^H-^1^H COSY correlations between 2H-10 and 3H-14 indicated that a partially hydrogenated olefinic carbon chain was present. In fragment II, the HMBC correlations from H-1′ (δ_H_ 2.92) to C-3′ (δ_C_ 195.1), C-6′ (δ_C_ 78.7), C-9′ (δ_C_ 193.1), C-10′ (δ_C_ 57.6), CH_3_-6′ (δ_C_ 21.1), and CH_3_-10′ (δ_C_ 18.2), and from H-7′ (δ_H_ 3.00) to C-3′, C-4′ (δ_C_ 57.9), C-5′ (δ_C_ 103.9), C-12′ (δ_C_ 78.8), C-9′, CH_3_-4′ (δ_C_ 19.0), and CH_3_-12′ (δ_C_ 21.3), revealed that the sorbicillinoid monomer II had an open-ended cage structure (Gao et al., 1995). The ^1^H-^1^H COSY correlations between 2H-14′ and 3H-18′ also indicated that there was a partially hydrogenated olefinic carbon chain. The key HMBC correlations from H-8 (δ_H_ 3.61) to C-7 (δ_C_ 50.6) and C-18 (δ_C_ 190.6) and from H-1′ (δ_H_ 2.92) to C-18 verified the oxygen-bearing carbon atom C-18 linked between two sorbicillinone moieties (Supplementary Figure 44).

**FIGURE 3:**
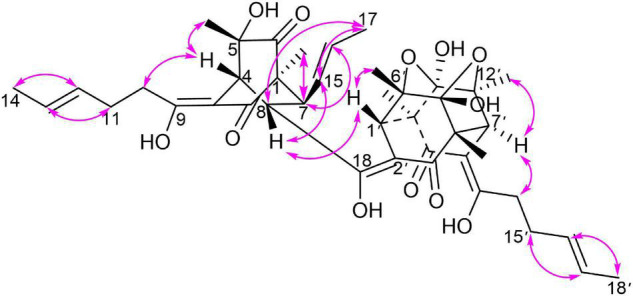
Key NOESY correlations of compound **1**.

Monomer I was a [2,2,2] octane group, and its relative configuration was established by NOE interactions (Figure 3). On the basis of the NOESY analysis, the cross-peaks of H-8 with H-1′ and H-15 revealed that H-8, H-1′ and H-15 should be on the same side of the molecule, suggesting that Δ^18^ had a *Z* configuration. The key NOE correlation observed between H-7 and CH_3_-1 indicated that they existed in a *cis* relationship. NOESY correlations of H-4 with CH_3_-5 and 2H-10 indicated that they all existed on the same side of the bicyclo [2,2,2] octane moiety (Li et al., 2007). These comparisons allowed us to presume the relative configuration of monomer I in **1** as 1S*,4R*,5S*,7S*,8S*. In monomer II, The NOESY correlations were all matched with those of dihydrotrichodimerol (Liu et al., 2005a). In addition, the NOESY correlations of H-4 with 2H-10 and of H-7′ with H-14′ indicated that Δ^3^ and Δ^8′^ had *Z* configurations. The double bond *E* configurations in the three open carbon chains were deduced by the large coupling constant (^3^*J*_15,16_ = 14.6 Hz) and by the NOESY correlations between H-11 and H-13, H-15 and H-17, and H-15′ and H-17′.

**FIGURE 4:**
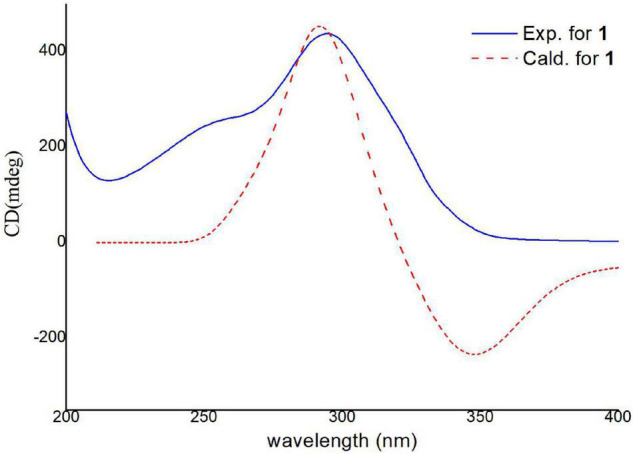
Experimental and calculated ECD spectra of compound **1** (calcd. ECD curves with a bathochromic shift for 20 nm).

Electronic circular dichroism (ECD) calculations were further employed to determine the absolute configuration of **1** (Supplementary Figure 2). The predicted ECD spectrum was obtained by the TDDFT [mPW1PW91/6–311G(d)] method and was subsequently compared with the experimental data. The calculated ECD spectrum agreed with the experimental curve, confirming the absolute configuration of compound **1** as 1*S*,4*R*,5*S*,7*S*,8*S*,1′*S*,4′*R*,5′*R*,6′*S*,7′*S*,10′*R*,11′*R*,12′*S* (Figure 4).

For the six reported trisorbicillinoids (Li et al., 2007, 2010; Guo et al., 2013; Cao et al., 2020), trisorbicillinones A, B, C, and D, 10,11,27,28-tetrahydrotrisorbicillinone C, and sorbicillamine E (Supplementary Figure 1), it was not possible to deduce the absolute relations of the whole molecule. The reason was that the two sorbicillinone fragments had long distance and the complex chiral centers. Nevertheless, in compound **1**, there was only one carbon atom between two sorbicillinone fragments, and NOESY correlations could be detected within the limits. Hence, combined with the ECD data, trisorbicillinone E (**1**) was the first trisorbicillinoid compound with the full absolute configuration determined.

**FIGURE 5:**
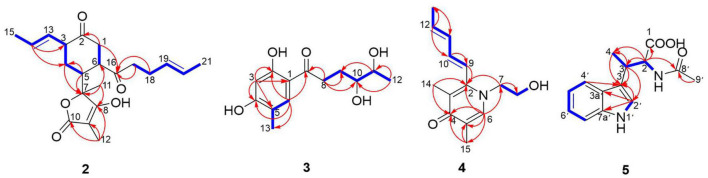
^1^H-^1^H COSY (blue bold lines), Key HMBC (red arrows) correlations of compounds **2**-**5**.

Acremosorbicillinoid A (**2**) was isolated as a yellow oil and had a molecular formula of C_21_H_28_O_5_, as deduced from its positive HRESIMS ion at *m/z* 383.1833 [M + Na]^+^ (Supplementary Figure 20), indicating eight degrees of unsaturation. Its IR absorptions at 3,495 and 1,725 cm^–1^ (Supplementary Figure 21) indicated that hydroxyl and carbonyl groups were present. The ^1^H NMR spectrum of **2** (Table 2 and Supplementary Figure 12) revealed four methyl proton signals, four methylene proton signals, seven methine signals including four olefinic signals at 5.22–5.35 ppm, and three aliphatic signals at 2.25–2.92 ppm. In the ^13^C NMR spectrum (Table 2 and Supplementary Figure 13), there were six quaternary carbons. Based on the MS requirements, these data suggested that two rings were present in compound **2**. The ^1^H-^1^H COSY correlations (Figure 5) between 3H-21/2H-17 and between H-13/3H-15 were assigned to two unsaturated chains. The key HMBC spectrum (Figure 5) of 3H-12 (δ_H_ 1.34) with C-10 (δ_C_ 177.6), C-9 (δ_C_ 81.0), and C-8 (δ_C_ 131.6) was expected to be an α,β-unsaturated lactone. The signals from 3H-11 (δ_H_ 1.06) to C-8, C-7 (δ_C_ 83.3), and C-6 (δ_C_ 40.0), from H-5 (δ_H_ 2.92) to C-7 and C-4 (δ_C_ 41.0), and from 2H-4 (δ_H_ 2.35, 2.05) to C-7 revealed that the α,β-unsaturated lactone was located at C-7. The HMBC correlations from 2H-1 (δ_H_ 2.38, 2.33) to C-2 (δ_C_ 210.5) and C-16 (δ_C_ 212.8), from 2H-17 (δ_H_ 2.27) and 2H-18 (δ_H_ 2.04) to C-16, and from H-14 (δ_H_ 5.31) to C-3 (δ_C_ 37.7) and C-4 were assigned to a six-membered ketone, with two sorbyl side chains.

**FIGURE 6:**
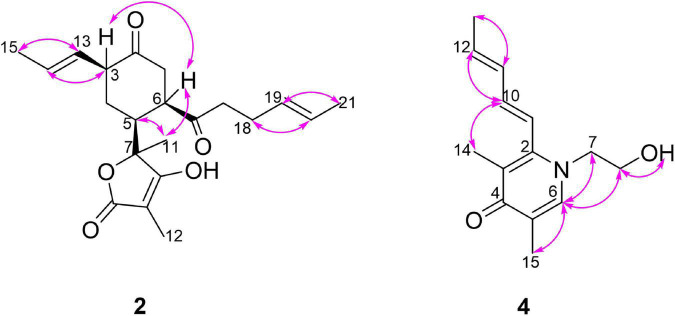
Key NOESY and 1D NOE correlations of compounds **2** and **4**.

**FIGURE 7:**
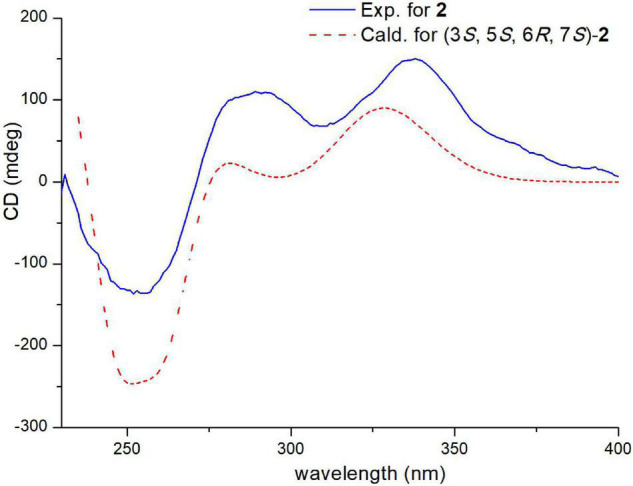
Experimental and calculated ECD spectra of compound **2** (calcd. ECD curves with a bathochromic shift for 45 nm).

The relative configuration of **2** was deduced by the coupling constants and NOESY spectrum (Figure 6). The large coupling constants (^3^*J*_13,14_ = 15.4 Hz) and NOESY correlations (Supplementary Figure 17) between H-13 and H-15, between H-18 and H-20, and between H-19 and H-21 indicated that the Δ^13^ and Δ^19^ double bonds had *E* configurations. The 1D NOE correlations (Supplementary Figure 18) of H-11 with H-5 and H-6 confirmed that they had the same orientation. In addition, in the ^1^H NMR spectrum, ^3^*J*_5,6_ = 0 Hz eliminated the pseudoaxial orientation of H-5 and H-6 (Isaka et al., 2001). The NOE cross-peak (Supplementary Figure 19) between H-3/H-6 revealed that H-3 and H-6 were on the same side. The relative configuration of the four stereocenters of **2** was determined to be 3S*,5S*,6R*,7S*. Furthermore, ECD calculations were employed to determine the absolute configuration (Supplementary Figure 2). The calculated ECD spectrum of **2** was obtained by the TDDFT [mPW1PW91/6–311G(d)] method and was subsequently compared with the experimental spectrum (Figure 7). The results revealed that the absolute configuration of compound **2** was 3*S*,5*S*,6*R*,7*S.*

Acremosorbicillinoid B (**3**), a yellow oil, was determined to have a molecular formula of C_13_H_18_O_5_, with five indices of hydrogen deficiency based on HRESIMS analysis (ion peak at *m/z* 277.1053 [M + Na]^+^) (Supplementary Figure 27). Its IR spectrum displayed absorptions that were attributed to hydroxy (3,495 cm^–1^), carbonyl (1,724 cm^–1^), and aromatic (1,594, 1,425 cm^–1^) groups. The ^1^H NMR data (Table 3) displayed signals for two para-oriented aromatic singlet hydrogens (δ_H_ 6.29, 7.61), two methylene groups (δ_H_ 3.03, 2.94, 1.89, 1.53), two oxygenated methine groups (δ_H_ 3.21, 3.40), and two methyl signals (δ_H_ 1.06, 2.05). The ^13^C NMR and HSQC data resolved 13 carbon signals attributable to six aromatic carbons (δ_C_ 101.9-164.0), including two oxygenated aromatic carbon atoms (δ_C_ 164.0, 162.8). One carbonyl group (δ_C_ 204.9), two oxygenated secondary carbons (δ_C_ 74.2, 69.6), two methylene groups (δ_C_ 34.0, 28.2), and two methyl carbons (δ_C_ 19.5, 15.3) were also observed. A carbochain with two hydroxy groups, located at C-10 and C-11, was established according to the HMBC correlations (Figure 5) from 2H-8 (δ_H_ 3.03, 2.94) to C-7 (δ_C_ 204.9), C-9 (δ_C_ 28.2), and C-10 (δ_C_ 74.2), from 3H-12 (δ_H_ 1.06) to C-11 (δ_C_ 69.6), and C-10, and from H-11 (δ_H_ 3.40) to C-9, as well as the 2H-8/2H-9/H-10/H-11/3H-12 spin system observed in the ^1^H-^1^H COSY spectrum. Additionally, the HMBC signals from H-6 (δ_H_ 7.61) to C-2 (δ_C_ 164.0), C-4 (δ_C_ 162.8), C-7, and C-13 (δ_C_ 15.3) and from H-3 (δ_H_ 6.29) to C-1 (δ_C_ 111.5), C-2, C-4, and C-5 (δ_C_ 116.6) indicated the four replacement positions of the benzene derivatives.

**FIGURE 8:**
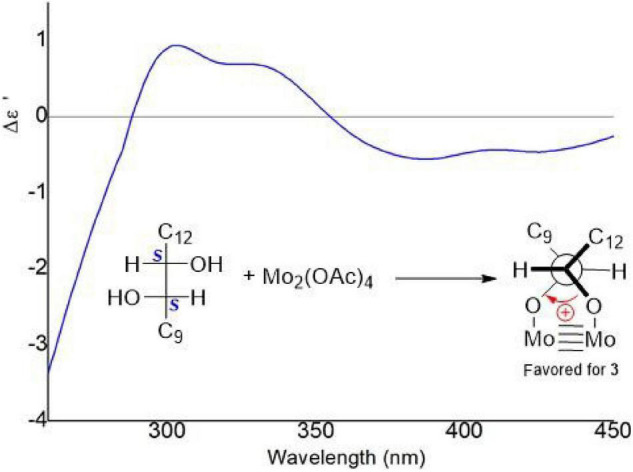
Induced circular dichroism (ICD) spectra from the Mo_2_-complexe of **3** in DMSO.

The vicinal diolsabsolute configurations in **3** were revealed by dimolybdenum tetraacetate [Mo_2_ (OAc)_4_]-induced CD (ICD, Snatzke’s method). In the ICD spectra, the Mo_2_ complex of **3** in DMSO gave a positive Cotton effect at approximately 310 nm (Figure 8). By the helicity rule of the Snatzke’s method, the torsional angle sign exhibited the signs of particular Cotton effects (Di Bari et al., 2001; Frelek et al., 2008), and the positive sign of the band was determined by the clockwise O–C–C–O torsional angle (the positive sign of the torsional angle). Since the coupling of H-10 and H-11 (^3^*J* = 6.18 Hz) indicated that the 10,11-diols in **3** had *threo* relative configuration (Jarvis et al., 1982, 1987), the conformation in the Mo_2_-complexe of **3** was preferred as determined by the ICD investigation (Figure 8). Thus, the absolute configurations of **3** at C-10 and C-11 could be assigned as 10*S* and 11*S*, respectively. Acremosorbicilliooid B (**3**) is a monomeric sorbicillinoid compound that is similar to sorbicillin (Supplementary Figure 43), and sorbicillin is the first member of the sorbicillinoid family (Cram, 1948; Cram and Tishler, 1948).

Acremosorbicillinoid C (**4**) was obtained as yellow oil. The molecular formula C_14_H_19_NO_2_ was established by the positive HRESIMS ion at *m/z* 234.1485 [M + H]^+^ (Supplementary Figure 35). The IR absorption at approximately 3,495 cm^–1^ was attributed to hydroxyl groups, while the peaks at 1,632 and 1,534 cm^–1^ implied the presence of ketones. Six degrees of unsaturation were indicated in addition to the ^1^H and ^13^C NMR data (Table 4). The ^1^H NMR spectrum of **4** displayed two singlet methyl signals, one doublet methyl signal, two oxygenated or nitrogenated methylene proton signals, five olefinic methine proton signals, and an exchangeable hydrogen proton. The ^13^C NMR spectrum showed fourteen carbons including one carbonyl signal, eight olefinic carbons, two methylenes, and three methyls. As one carbonyl and eight olephinic carbons accounted for five degrees of unsaturation, compound **4** was inferred to have a ring structure. Based on the ^1^H-^1^H COSY data, the correlative signal (Figure 5) between H-9 and 3H-13 suggested that a hydrogenated olefinic carbon chain was present. The key HMBC signal from H-9 (δ_H_ 6.36) to C-2 (δ_C_ 144.4) indicated that the pentadiene group fragment should be connected to C-2. The positions of two methyls, one carbonyl unit and one ethoxy, were established by the key HMBC connections of 3H-14/C-2 and C-4, of 3H-15/C-4, and C-5, of H-6/C-2, C-4, C-5, C-7 and C-15, and of 2H-7/C-2 and C-8. The coupling constants (^3^*J*_9,10_ = 15.9 Hz, ^3^*J*_11,12_ = 15.3 Hz), and the NOESY correlation (Figure 6) between H-11 and H-13 indicated that the two ethylenic bonds in the pentadiene group had *E* configurations.

Acetyl-β-methyltryptophan (**5**) was obtained as a colorless needle crystal. Its molecular formula was determined to be C_14_H_16_N_2_O_3_ according to the HRESIMS peak at *m/z* 261.1234 [M + H]^+^ (Supplementary Figure 42). The combined 1D NMR spectroscopic data (Table 5) showed that compound **5** contained two carbonyl groups (C-1 and C-8′), two methyl groups (C-4 and C-9′), and an indol ring. The key HMBC connections (Figure 5) of H-2′/C-3′, H-2′/C-3a′, H-2′/C-7a′, and H-4′/C-3′ confirmed the existence of the indole moiety. The key HMBC correlations from H-2 (δ_H_ 4.57) and H-9′ (δ_H_ 1.83) to C-8′ (δ_C_ 169.2), from H-2 to C-1 (δ_C_ 173.6), C-3 (δ_C_ 32.3), and C-4 (δ_C_ 16.6), and from H-3 (δ_H_ 3.57) to C-4 indicated an amide carbon chain group was present in compound **5**. The side chain connection point was C-3′, which was deduced by the HMBC correlations from H-3 to C-2′ (δ_C_ 122.2) and C-3′ (δ_C_ 116.4), and from H-4 to C-3′.

**FIGURE 9:**
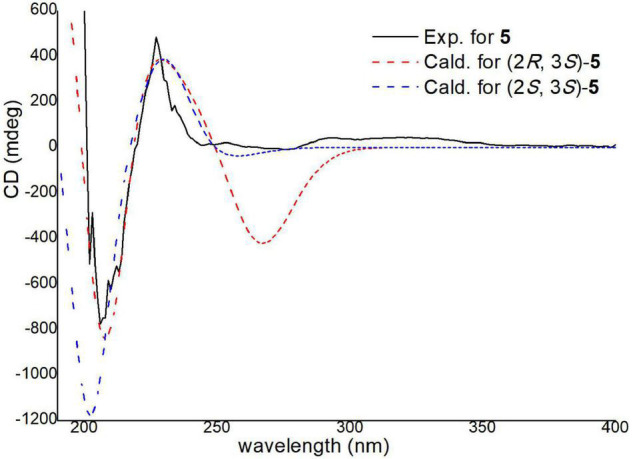
Experimental and calculated ECD spectra of compound **5**.

The absolute configurations of C-2 and C-3 were determined by ECD calculations (Supplementary Figure 2). The predicted ECD spectra of 2*S*,3*R*-**5** and 2*S*,3*S*-**5** were obtained by the TDDFT [mPW1PW91/6–311G(d)] method, and were compared with the experimental data (Figure 9). The 2*S*,3*S* calculated ECD spectrum agreed well with the experimental curve, confirming the absolute configuration of compound **5** as 2*S*,3*S*. Acetyl-β-methyltryptophan was recorded as an intermediate product to synthetize β-methyltryptophan (Snyder and Matteson, 1957). Moreover, 2*S*,3*S*-acetyl-β-methyltryptophan (**5**) was first obtained as a natural product from a natural source.

The structures of known compounds **6–13** were established by comparing their NMR data with previously reported data (Andrade et al., 1992; Shirota et al., 1997; Liu et al., 2005b,c; Yu et al., 2019). In addition, crystals of penicillones B (**13**) that were suitable for X-ray diffraction were obtained from the solvent CH_3_OH. A single-crystal X-ray crystallographic analysis of **13** conducted with Cu Kα radiation [Flack parameter of −0.02 (15)] (Supplementary Figure 45) first determined the challenging absolute configuration of **13**.

### Activity

None of the five new compounds (**1**-**5**) exhibited cytotoxic effects against the five human tumor cell lines and HBE cell line (IC_50_ > 40 μM), or any antimicrobial activities (MIC > 4 μg/well). None of the thirteen compounds (**1**-**13**) showed antifungal activities against agricultural pathogenic fungi (MIC > 40 μg/well), or QS inhibitory activity against *C. violaceum* CV026 (MIC > 40 μg/well). Know compound **11** exhibited cholesterol efflux enhancing activity (Supplementary Figure 46).

## Conclusion

In this study, three new sorbicillinoids, trisorbicillinone E (**1**), acremosorbicillinoids A (**2**) and B (**3**), and two new compounds, acremokaloid A (**4**), and 2*S*,3*S*-acetyl-β-methyltryptophan (**5**), together with eight known sorbicillinoids (**6***-***13**), were isolated from the endophytic fungus *A. citrinum* SS-g13. Extensive NMR spectroscopic analysis and ECD calculations were used to elucidate the structures of the new molecules, including their absolute configurations. Compound **1**, as the first trisorbicillinoid compound with an established absolute configuration, belongs to the trimeric sorbicillinoids family and is rare in nature. A plausible biosynthesis pathway with monomeric sorbicillinoid, bisorbicillinoid, trisorbicillinoid, and hybrid sorbicillinoid, four different structural types is proposed in Scheme 1. Sorbicillinol was hypothesized as a precursor of most sorbicillinoids. Sorbicillinoids were hypothesized as biosynthesized by polyketide synthases (PKs), and were proposed to be synthesized through intermolecular Diels-Alder, Michael dimerization reactions, and epoxidation. These theories are supported by literature precedence (Harned and Volp, 2011; Pang et al., 2021).

**SCHEME:**
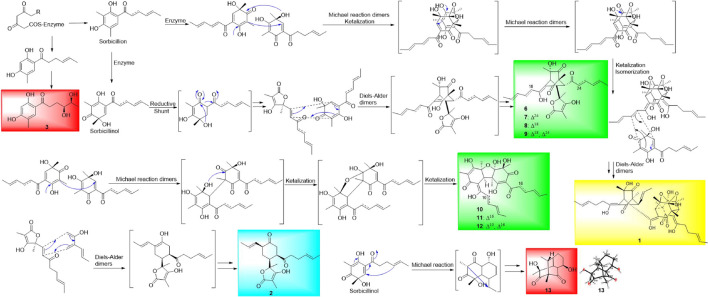
Plausible Biosynthetic Pathways of Monomeric Sorbicillinoids (red shaded), Bisorbicillinoids (green shaded), Trisorbicillinoid (yellow shaded), and Hybrid Sorbicillinoid (blue shaded).

## Data Availability Statement

The datasets presented in this study can be found in online repositories. The names of the repository/repositories and accession number(s) can be found in the article/Supplementary Material.

## Author Contributions

X-PP performed the isolation, purification, and structural characterization of all the compounds, and prepared the manuscript. GL and PW performed the ECD calculations. CW and QW contributed to the isolation of compounds and revised the manuscript. L-MW, Z-QH, and L-XJ contributed to the bioactivity evaluation. G-FL contributed to the physical constant determination of compounds. Z-YJ and C-XC contributed to the isolation and identification of the fungal strain. H-XL designed the research, and revised the manuscript. All authors have read and agreed to the published version of the manuscript.

## Conflict of Interest

The authors declare that the research was conducted in the absence of any commercial or financial relationships that could be construed as a potential conflict of interest.

## Publisher’s Note

All claims expressed in this article are solely those of the authors and do not necessarily represent those of their affiliated organizations, or those of the publisher, the editors and the reviewers. Any product that may be evaluated in this article, or claim that may be made by its manufacturer, is not guaranteed or endorsed by the publisher.
