# Supplementary material for: Structurally Various Sorbicillinoids From an Endophytic Fungus Acremonium citrinum SS-g13
Source: Front Microbiol. 2022 Mar 23;13:800626. doi: 10.3389/fmicb.2022.800626 (PMC8997241; doi:10.3389/fmicb.2022.800626)
Supplement: Supplementary file 1 [file Data_Sheet_1.PDF]

Supporting Information

New Trimeric, Hybrid, Monomeric Sorbicillinoids from an Endophytic Fungus *Acremonium citrinum* SS-g13

Xiao-Ping Peng[1], Gang Li[1], Li-Mei Wang[1], Qi Wang[1], Cong Wang[3], Li-Xia Ji[1], Chen-Xi Cao[1], Guo-Feng Lin[1], Zu-Yang Jiang[1], Zhuo-qian He[1], Pei Wang[4], and Hong-Xiang Lou[1,2]*

[1]Department of Natural Medicinal Chemistry and Pharmacognosy, School of Pharmacy, Qingdao University, Qingdao 266071, China

[2]Key Laboratory of Chemical Biology of Ministry of Education, Department of Natural Product Chemistry, School of Pharmaceutical Sciences, Shandong University, Jinan 250012, China

[3]Guangxi Key Laboratory of Chemistry and Engineering of Forest Products, Guangxi University for Nationalities, Nanning 530006, China

[4]Hainan Key Laboratory of Research and Development of Natural Product from Li Folk Medicine, Hainan Institute for Tropical Agricultural Resources, Institute of Tropical Bioscience and Biotechnology, Chinese Academy of Tropical Agricultural Sciences, Haikou 571101, China

***Correspondence:**

Hongxiang Lou

louhongxiang@sdu.edu.cn

Contents	page
**Table S1.** NMR data for compound **1**	4
**Table S2.** NMR data for compound **2**	6
**Table S3.** NMR data for compound **3**	7
**Table S4.** NMR data for compound **4**	8
**Table S5.** NMR data for compound **5**	9
**Table S6.** Gibbs free energies and equilibrium populations of low-energy conformers of **1**	10
**Table S7.** Gibbs free energies and equilibrium populations of low-energy conformers of **2**	10
**Table S8.** Gibbs free energies and equilibrium populations of low-energy conformers of (2*R*, 3*R*)-**5**	11
**Table S9.** Gibbs free energies and equilibrium populations of low-energy conformers of (2*S*, 3*R*)-**5**	12
**Figure S1.** Typical structures of four groups sorbicillinoids	13
**Figure S2.** Computational details of compounds **1**, **2** and **5**	14
**Figure S3.** The 18S rRNA gene sequences data of *Acremonium citrinum* SS-g13	15
**Figure S4.** The ^{1}H NMR (500 MHz, $CDCl_3$) spectrum of compound **1**	16
**Figure S5.** The ^{13}C NMR (125 MHz, $CDCl_3$) spectrum of compound **1**	16
**Figure S6.** The HSQC (500 MHz, $CDCl_3$) spectrum of compound **1**	17
**Figure S7.** The ^{1}H-^{1}H COSY (500 MHz, $CDCl_3$) spectrum of compound **1**	17
**Figure S8.** The HMBC (500 MHz, $CDCl_3$) spectrum of compound **1**	18
**Figure S9.** The NOESY (500 MHz, $CDCl_3$) spectrum of compound **1**	18
**Figure S10.** The HRESIMS spectrum of compound **1**	19
**Figure S11.** IR spectrum of compound **1**	19
**Figure S12.** The ^{1}H NMR (500 MHz, DMSO-d_6) spectrum of compound **2**	20
**Figure S13.** The ^{13}C NMR (125 MHz, DMSO-d_6) spectrum of compound **2**	20
**Figure S14.** The HSQC (500 MHz, DMSO-d_6) spectrum of compound **2**	21
**Figure S15** The ^{1}H-^{1}H COSY (500 MHz, DMSO-d_6) spectrum of compound **2**	21
**Figure S16.** The HMBC (500 MHz, DMSO-d_6) spectrum of compound **2**	22
**Figure S17.** The NOESY (500 MHz, DMSO-d_6) spectrum of compound **2**	22

Figure S18. The 1D NOE (500 MHz, DMSO-d_6) spectrum of compound **2** (1) 23
Figure S19. The 1D NOE (500 MHz, DMSO-d_6) spectrum of compound **2** (2) 23
Figure S20. The HRESIMS spectrum of compound **2** 24
Figure S21. IR spectrum of compound **2** 24
Figure S22. The ^{1}H NMR (600 MHz, DMSO-d_6) spectrum of compound **3** 25
Figure S23. The ^{13}C NMR (150 MHz, DMSO-d_6) spectrum of compound **3** 25
Figure S24. The HSQC (500 MHz, DMSO-d_6) spectrum of compound **3** 26
Figure S25. The ^{1}H-^{1}H COSY (500 MHz, DMSO-d_6) spectrum of compound **3** 26
Figure S26. The HMBC (500 MHz, DMSO-d_6) spectrum of compound **3** 27
Figure S27. The HRESIMS spectrum of compound **3** 27
Figure S28. IR spectrum of compound **3** 28
Figure S29. The ^{1}H NMR (500 MHz, DMSO-d_6) spectrum of compound **4** 28
Figure S30. The ^{13}C NMR (125 MHz, DMSO-d_6) spectrum of compound **4** 29
Figure S31. The HSQC (500 MHz, DMSO-d_6) spectrum of compound **4** 29
Figure S32. The ^{1}H-^{1}H COSY (500 MHz, DMSO-d_6) spectrum of compound **4** 30
Figure S33. The HMBC (500 MHz, DMSO-d_6) spectrum of compound **4** 30
Figure S34. The NOESY (500 MHz, DMSO-d_6) spectrum of compound **4** 31
Figure S35. The HRESIMS spectrum of compound **4** 31
Figure S36. IR spectrum of compound **4** 32
Figure S37. The ^{1}H NMR (600 MHz, DMSO-d_6) spectrum of compound **5** 32
Figure S38. The ^{13}C NMR (150 MHz, DMSO-d_6) spectrum of compound **5** 33
Figure S39. The HSQC (400 MHz, DMSO-d_6) spectrum of compound **5** 33
Figure S40. The ^{1}H-^{1}H COSY (400 MHz, DMSO-d_6) spectrum of compound **5** 34
Figure S41. The HMBC (400 MHz, DMSO-d_6) spectrum of compound **5** 34
Figure S42. The HRESIMS spectrum of compound **5** 35
Figure S43. The structures of trisorbicillinone D and sorbicillin 36
Figure S44. ORTEP diagram for the single-crystal X-ray geometry of **13** 36
Figure S45. Influence of compounds **6**, **7**, **8**, **11**, and **12** on serum-mediated cholesterol efflux and on cell viability in J774A.1 macrophages. 37
References 39

Table S1. NMR data for compound **1**.

position	δ_C, type	δ_H, (J in Hz)	HMBC (H→C)	COSY
1	62.3, C			
2	195.7, C			
3	107.8, C			
4	45.6, CH	3.04, s	2, 3, 5, 9	8
5	75.8, C			
6	211.4, C			
7	50.6, CH	2.75, m	6	8, 15
8	41.9, CH	3.61, d, (6.9)	3, 4, 5, 7, 15, 18	4, 7
9	181.8, C			
10	31.9, CH_2	2.16, m	9, 11, 12	11
11	28.5, CH_2	2.28, m	9	10, 12
12	129.3, CH	5.41, m		11, 13
13	126.8, CH	5.50, m	11, 14	12, 14
14	18.0, CH_3	1.66 [a], d, (9.2)	12	13
15	127.8, CH	4.98, dd, (14.6, 9.6)		7, 16
16	130.4, CH	5.35, m	7, 17	15, 17
17	17.7, CH_3	1.59, d, (6.3)	15	16
18	190.6, C			
1′	57.8, CH	2.92, s	18, 3′, 6′, 9′, 10′, CH_3-6′, CH_3-10′	
2′	103.7, C			
3′	195.1, C			
4′	57.9, C			
5′	103.9, C			
6′	78.7, C			
7′	57.9, CH	3.0, s	3′, 4′, 5′, 9′, 12′, CH_3-4′, CH_3-12′	
8′	104.3, C			
9′	193.1, C			
10′	57.6, C			
11′	104.0, C			
12′	78.8, C			
13′	192.5, C			
14′	34.7, CH_2	2.43, m	13′, 15′	15′
15′	28.5, CH_2	2.28, m	13′	14′, 16′
16′	129.1, CH	5.41, m		15′, 17′
17′	126.7, CH	5.49, m	15′, 18′	16′, 18′
18′	18.0, CH_3	1.64 [a], d, (9.1)	17′	17′
CH_3-1	9.9, CH_3	1.11, s	1, 6, 7	
CH_3-5	24.9, CH_3	1.26, s	4, 6	
CH_3-4′	19.0, CH_3	1.42, s	3′	
CH_3-6′	21.1, CH_3	1.30, s	5′	

CH_3-10′	18.2, CH_3	1.52, s	11′
CH_3-12′	21.3, CH_3	1.44, s	
OH-5			
OH-9			
OH-5′			
OH-11′			
OH-13′			

[a] The assignments could be interchanged.

Table S2. NMR data for compound **2**.

position	δ_C, type	δ_H, (J in Hz)	HMBC (H→C)	COSY
1	37.9, CH_2	2.38, m 2.33, m	2, 16	
2	210.5, C			
3	37.7, CH	2.82, dt (6.9, 11.3)		4, 13
4	41.0, CH_2	2.35, m; 2.05, m	2, 3, 5, 7	3, 5
5	48.7, CH	2.92, t (4.9)	4, 6, 7, 16	4, 6
6	40.0, CH	2.25, m	1	
7	83.3, C			
8	131.6, C			
9	81.0, C			
10	177.6, C			
11	22.0, CH_3	1.06, s	6, 7, 8	
12	6.8, CH_3	1.34, s	8, 9, 10	
13	131.6, CH	5.22, dd (15.4, 7.3)	3	3, 14
14	125.3, CH	5.31, m	3, 4, 13, 15	13, 15
15	17.7, CH_3	1.56, d (6.8)	13, 14	14
16	212.8, C			
17	43.6, CH_2	2.27, m	16, 18, 19	14
18	25.8, CH_2	2.04, m	17, 19, 20	17, 19
19	130.3, CH	5.35, m		18, 20
20	124.7, CH	5.35, m		19, 21
21	17.6, CH_3	1.58, d (6.5)	19, 20	20

Table S3. NMR data for compound **3**.

position	δ_C, type	δ_H, (J in Hz)	HMBC (H→C)	COSY
1	111.5, C			
2	164.0, C			
3	101.9, CH	6.29, s	1, 2, 4, 5	
4	162.8, C			
5	116.6, C			
6	132.2, CH	7.61, s	2, 4, 7, 13	
7	204.9, C			
8	34.0, CH_2	3.03, m	7, 9, 10	9
		2.94, m		
9	28.2, CH_2	1.89, m		8, 10
		1.53, m		
10	74.2, CH	3.21, m ddd (2.9, 8.9, 6.2)		9, 11
11	69.6, CH	3.40, dq (6.2, 6.2)	9	10, 12
12	19.5, CH_3	1.06, d (6.2)	10, 11	11
13	15.3, CH_3	2.05, s	4, 5	

Table S4. NMR data for compound **4**.

position	δ_C, type	δ_H, (J in Hz)	HMBC (H→C)	COSY
2	144.4, C			
3	121.2, C			
4	176.7, C			
5	121.2, C			
6	138.5, CH	7.56, s	2, 4, 5, 7, 15	
7	55.0, CH_2	3.88, t (5.5)	2, 8	8
8	60.1, CH_2	3.56, m		7, 8-OH
9	121.3, CH	6.36, d (15.9)	2	10
10	138.8, CH	6.40, m	3, 9	9, 11
11	130.9, CH	6.29, dd (15.3, 7.4)		10, 12
12	133.2, CH	5.94, m	13	11, 13
13	18.1, CH_3	1.80, d (6.7)	11	12
14	13.3, CH_3	1.89, s	2, 4	
15	13.8, CH_3	1.84, s	4, 5	
8-OH		4.96, br s		8

Table S5. NMR data for compound **5**.

position	δ_C, type	δ_H, (J in Hz)	HMBC (H→C)	COSY
1	173.6, C			
2	57.2, CH	4.57, dd (8.6, 6.0)	1, 4, 3′, 8′	3
3	32.3, CH	3.57, m	2, 4, 2′, 3′	2, 4
4	16.6, CH_3	1.31, d (7.1)	2, 3, 3′	
1′		10.80, s		
2′	122.2, CH	7.12, s	3′, 3a′, 7a′	
3′	116.4, C			
3a′	126.5, C			
4′	118.4, CH	7.54, d (7.9)	3′, 6′, 3a′, 7a′,	5′
5′	118.1, CH	6.97, t (7.4)	7′, 3a′,	4′, 6′
6′	120.7, CH	7.05, t (7.5)	4′, 7a′	7′
7′	111.4, CH	7.32, d (8.1)	3a′	6′
7a′	136.1, C			
8′	169.2, C			
9′	22.5, CH_3	1.83, s	8′	
NH		7.94, d (8.8)		

Table S6. Gibbs free energies[a] and equilibrium populations[b] of low-energy conformers of **1**.

Conformers	In MeOH	
	G	*P* (%)
**1-1**	-2533.637046	100

[a]mPW1PW91/6-311G(d), in kcal/mol. [b]From *G* values at 298.15K.

Table S7. Gibbs free energies[a] and equilibrium populations[b] of low-energy conformers of **2**.

Conformers	In MeOH	
	G	*P* (%)
**2-1**	-959.530281	48.07
**2-2**	-959.530285	48.28
**2-3**	-959.527154	1.75
**2-4**	-959.524952	0.17
**2-5**	-959.527145	1.73

[a]mPW1PW91/6-311G(d), in kcal/mol. [b]From *G* values at 298.15K.

Table S8. Gibbs free energies[a] and equilibrium populations[b] of low-energy conformers of (2*R*, 3*R*)-**5**.

Conformers	In MeOH	
	G	*P* (%)
**5-1**	-878.132825	49.24
**5-2**	-878.126212	0.04
**5-3**	-878.132831	49.56
**5-4**	-878.125621	0.02
**5-5**	-878.129047	0.90
**5-6**	-878.123614	0.003
**5-7**	-878.127685	0.21
**5-8**	-878.124988	0.01
**5-9**	-878.123606	0.003
**5-10**	-878.11578	0.0000007

[a]mPW1PW91/6-311G(d), in kcal/mol. [b]From *G* values at 298.15K.

Table S9. Gibbs free energies[a] and equilibrium populations[b] of low-energy conformers of (2*S*, 3*R*)-**5**.

Conformers	In MeOH	
	G	*P* (%)
**5-1**	-878.132607	26.87
**5-2**	-878.130869	4.26
**5-3**	-878.130213	2.13
**5-4**	-878.13228	19.00
**5-5**	-878.130867	4.25
**5-6**	-878.131876	12.38
**5-7**	-878.132285	19.10
**5-8**	-878.125259	0.01
**5-9**	-878.12851	0.35
**5-10**	-878.131818	11.65
**5-11**	-878.128495	0.34
**5-12**	-878.130806	3.98
**5-13**	-878.130875	4.29
**5-14**	-878.130209	2.12
**5-15**	-878.12986	1.46
**5-16**	-878.130738	3.71
**5-17**	-878.125245	0.01
**5-18**	-878.130209	2.12
**5-19**	-878.128456	0.33
**5-20**	-878.128474	0.34
**5-21**	-878.126137	0.03
**5-22**	-878.128458	0.33
**5-23**	-878.130867	4.25
**5-24**	-878.129773	1.33
**5-25**	-878.130287	2.30
**5-26**	-878.130808	3.99

[a]mPW1PW91/6-311G(d), in kcal/mol. [b]From *G* values at 298.15K.

Monomeric Sorbicillinoids:

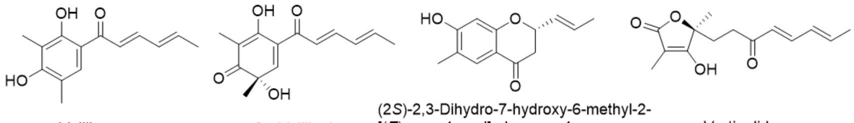

sorbicillin | Sorbicillinol | (2*S*)-2,3-Dihydro-7-hydroxy-6-methyl-2-[(*E*)-prop-1-enyl]-chroman-4-one | Vertinolide

Bisorbicillinoids:

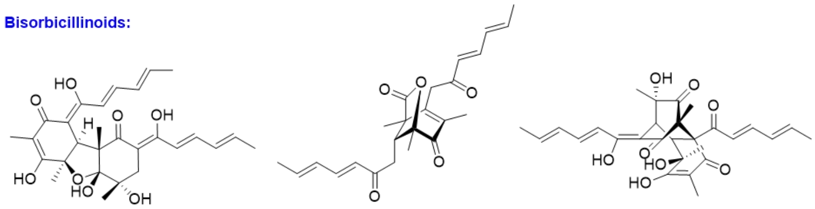

Isobisvertinol | Trichodermolide | Bisorbicillinol

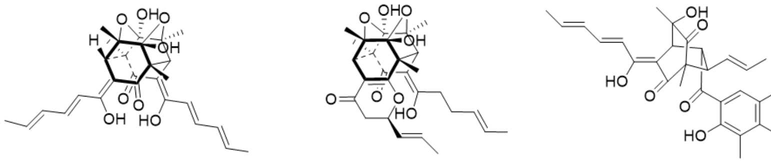

Trichodimerol | Tetrahydrotrichodimer ether | Sorbiquinol

Trisorbicillinoids:

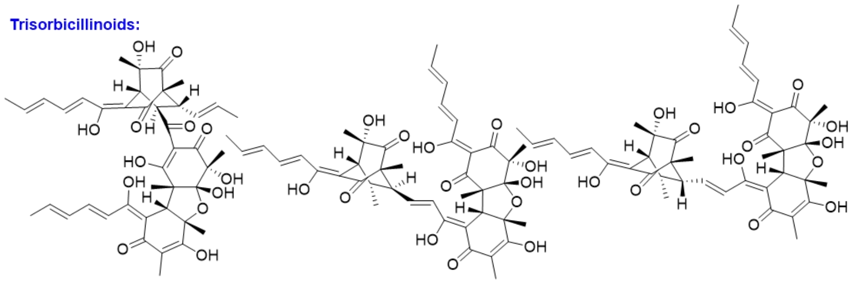

Trisorbicillinone A | Trisorbicillinone B | Trisorbicillinone C

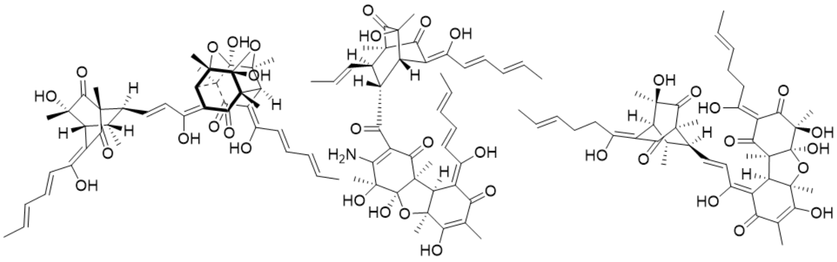

Trisorbicillinone D | Sorbicillamine E | 10,11,27,28-Tetrahydrotrisorbicillinone C

Hybrid Sorbicillinoids:

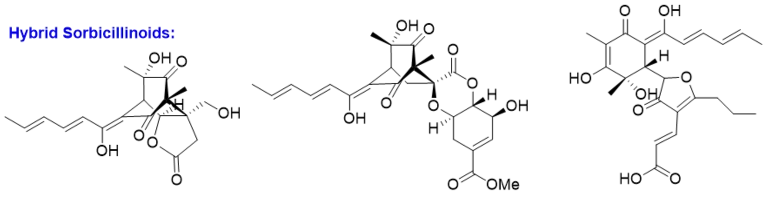

Rezishanone A | Spirosorbicillinol A | Sorbifuranone A

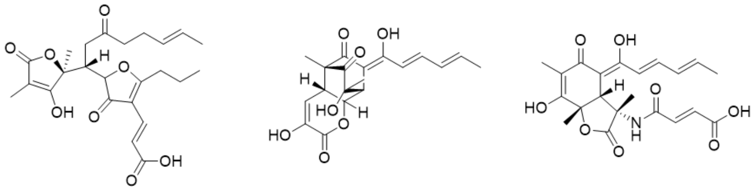

Sorbifuranone B | Trichodermanone D | Sorbicillactone A

Figure S1. Typical structures of four groups sorbicillinoids

ECD calculations for compounds **1**, **2** and **5** were performed as described in previous literature.[S1] The possible conformers were optimized using Frog2 online version.[S2] Further geometrical optimization and vibrational evaluation with Gaussian using DFT calculations with the B3PW91 functional and the TZVP basis set were carried out. All the DFT calculations were performed by Gaussian 16 software package with g09 defaults keyword. TDDFT at the mPW1PW91/6-311G(d) level of theory was applied to calculate the singlet electronic excitation energies and rotational strengths with the solvation PCM model with methanol. The ECD curve for each conformer was then simulated according to equation 4d to 6d with a half-band of 0.3 eV.[S3] The individual conformer values were summed for obtaining the final ECD spectrum with respect to their Boltzmann distributions.

Figure S2. Computational details of compounds **1**, **2** and **5**

TCCGTAGGGTGAACCTGCGGAGGGATCATTATAGAGTCTCTGAACTCCCAA
ACCTTTGTGAACATACCACTGTTGCTTCGGCGGCGTTCTGGGTGTCTCCGG
GCATCCAGGGTTCCACGCGCCGCCGAGGGTACCAAACTCTTGTCTTTATATC
GGACTTCTGAGTGAAAATACAAAATAAATTAAAACTTTCAGCAACGGATCT
CTTGGCTCTGGCATCGATGAAGAACGCAGCGAAATGCGATAAGTAATGTGA
ATTGCAGAATTCAGTGAATCATCGAATCTTTGAACGCACATTGCGCCCACC
AGTATTCTGGTGGGCATGCCTGTCCGAGCGTCATTTCAACCCTCAGGCCCA
GCCTGGTGTTGGGGATTGGCTTCGCGGCCACCCCCGAAATGCAGTGGCGG
CCCTCCGCGAACTCCTCTGTGCAGTAGTGATACCTCGCATTGGATAGTGGTT
GCGCCTCGCCGTAAAACCTCCAACTTCTCAAGGTTGACCTCGGATCAGGTA
GGAATACCCGCTGAACTTAAGCATATCAATAAGCGGAGAA

Figure S3. The 18S rRNA gene sequences data of *Acremonium citrinum* SS-g13

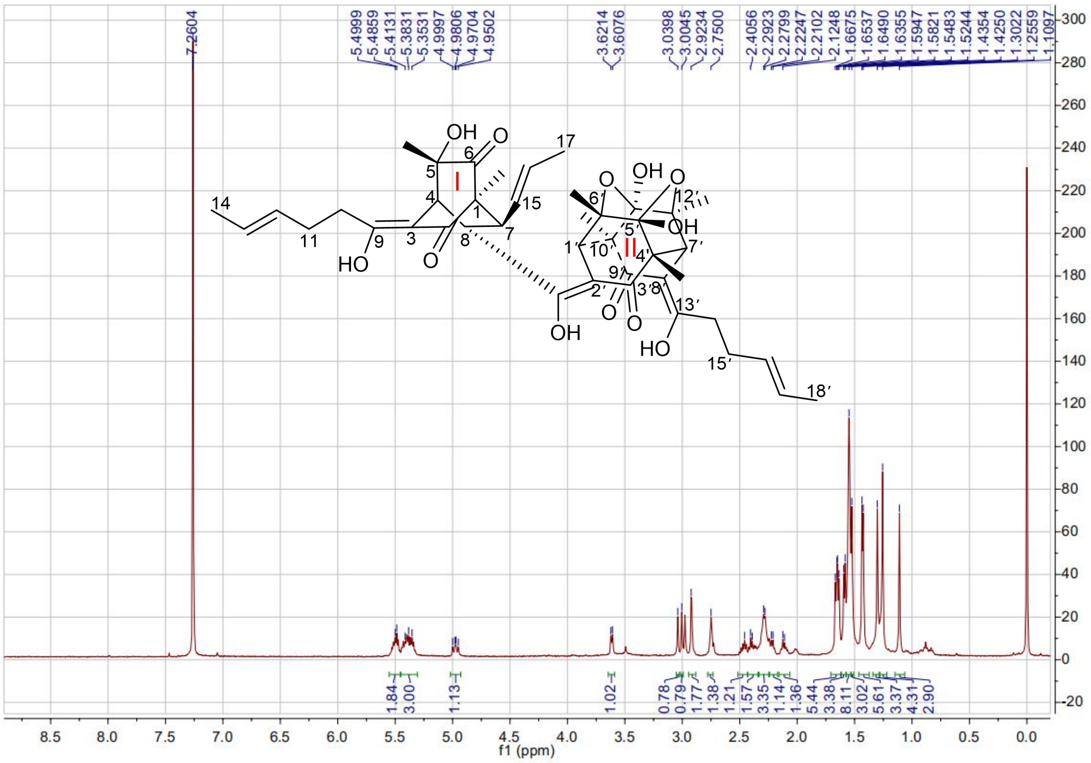

Figure S4 The ^{1}H NMR (500 MHz, $CDCl_3$-*d*) spectrum of compound **1**

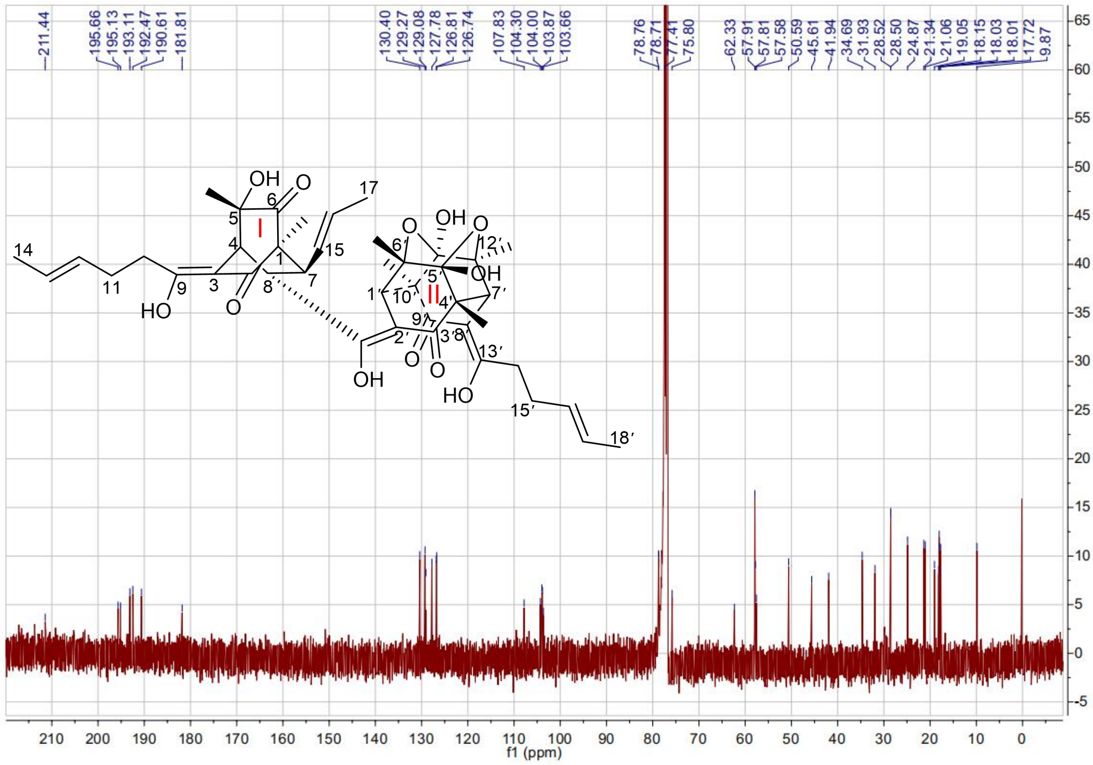

Figure S5 The ^{13}C NMR (125 MHz, $CDCl_3$-*d*) spectrum of compound **1**

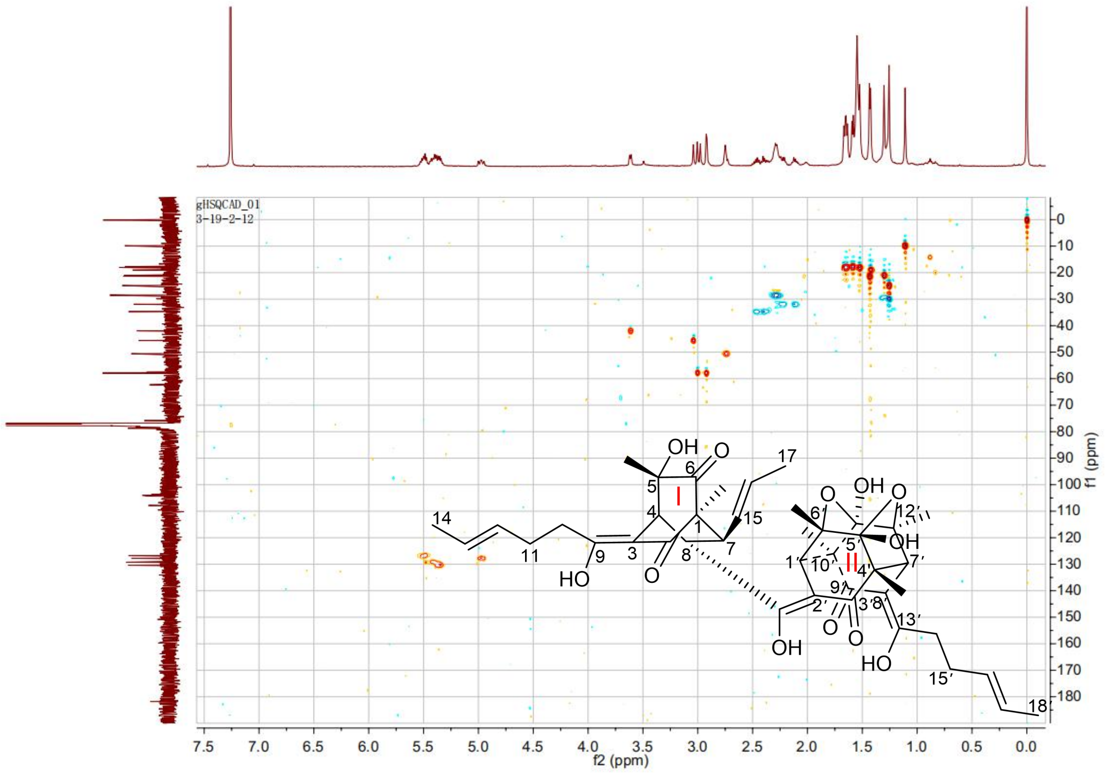

Figure S6 The HSQC (500 MHz, $CDCl_3$-*d*) spectrum of compound **1**

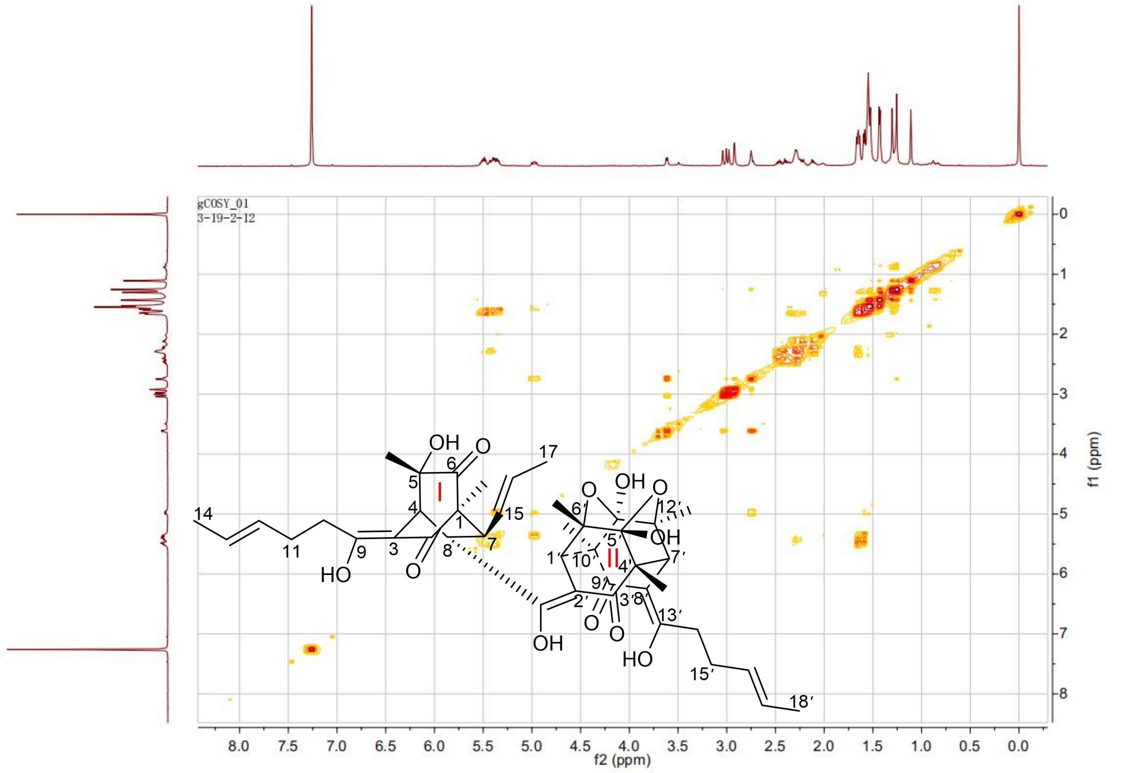

Figure S7 The ^1H-^1H COSY (500 MHz, $CDCl_3$-*d*) spectrum of compound **1**

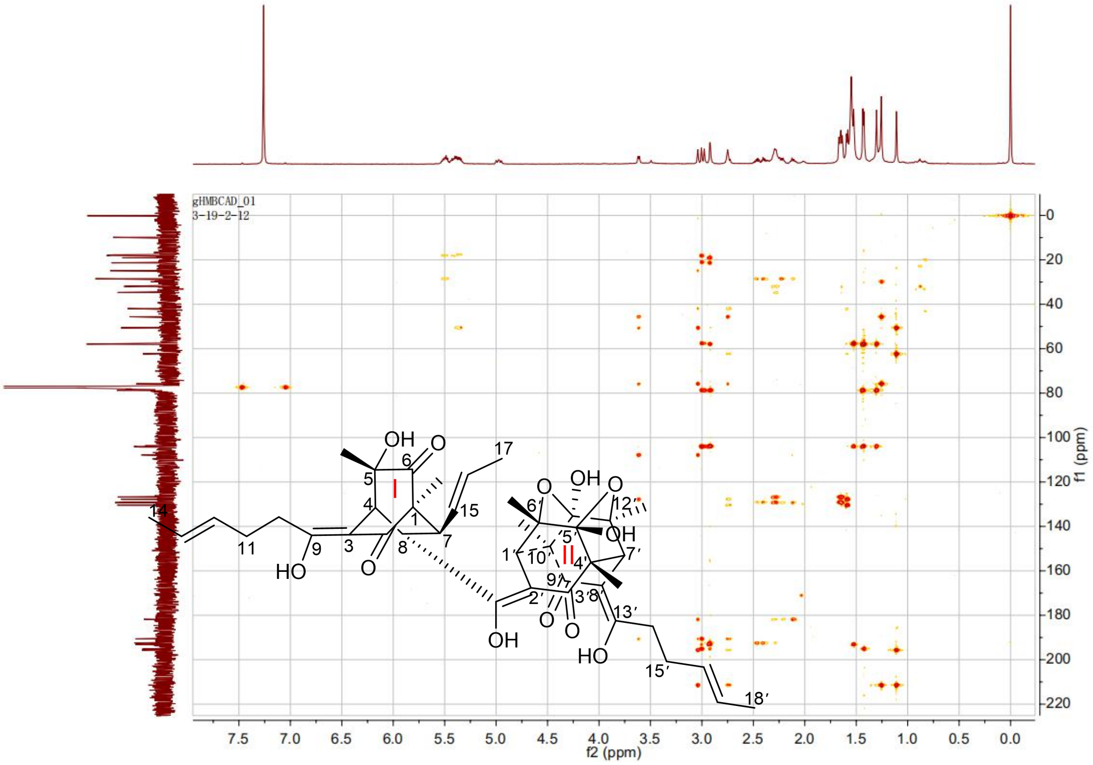

Figure S8 The HMBC (500 MHz, $CDCl_3$-*d*) spectrum of compound **1**

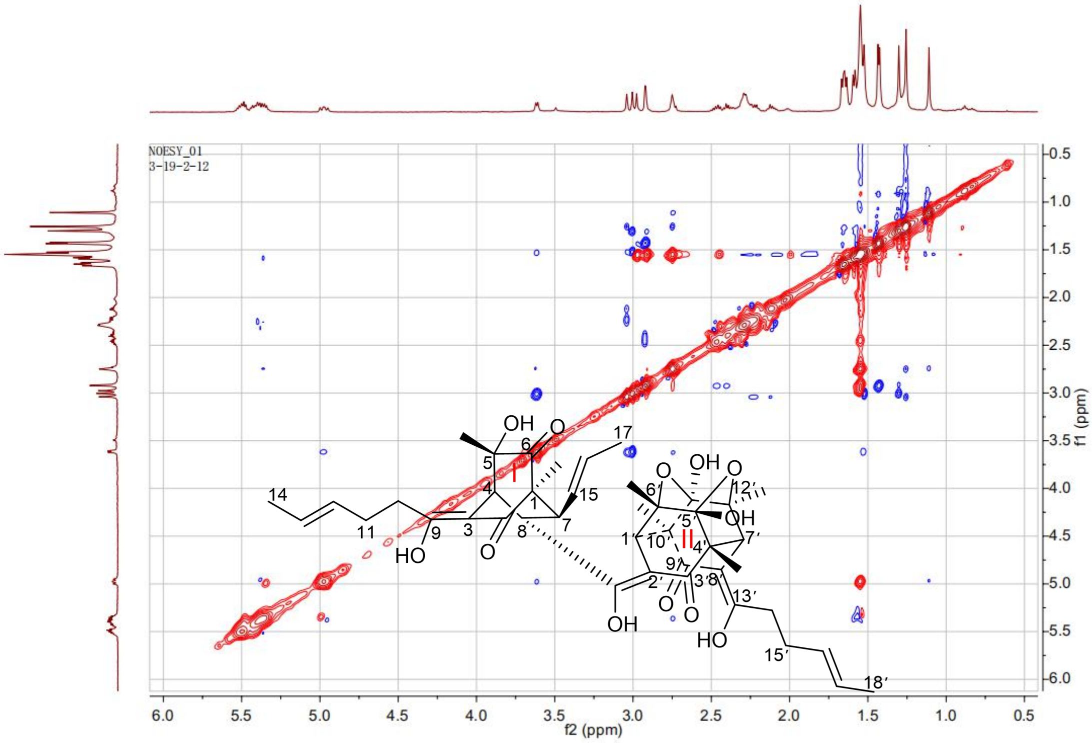

Figure S9 The NOESY (500 MHz, $CDCl_3$-*d*) spectrum of compound **1**

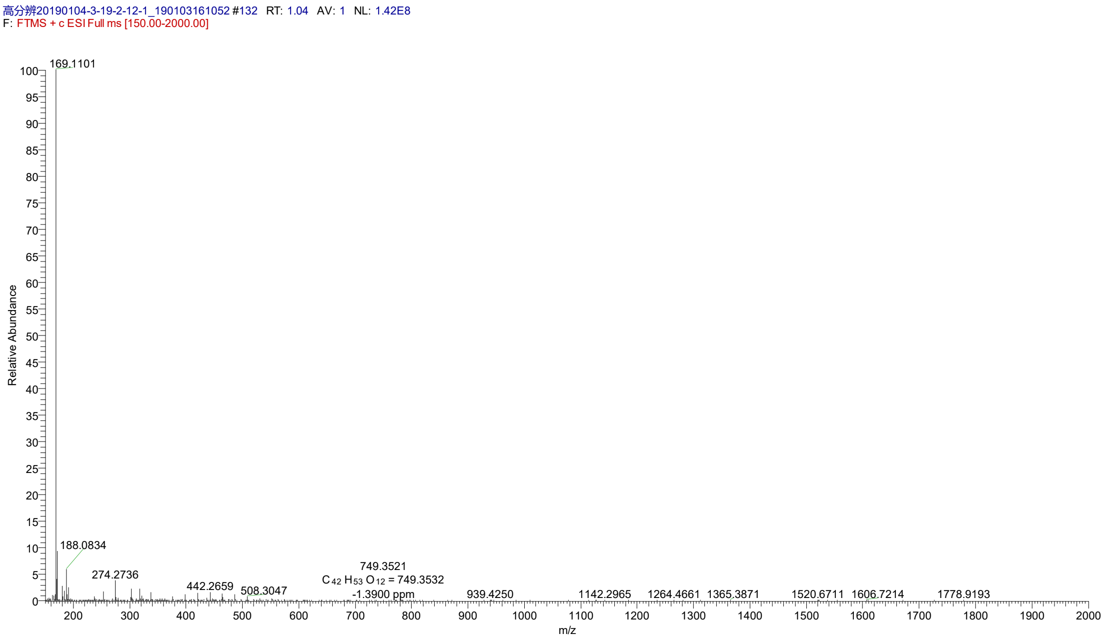

Figure S10 The HRESIMS spectrum of compound **1**

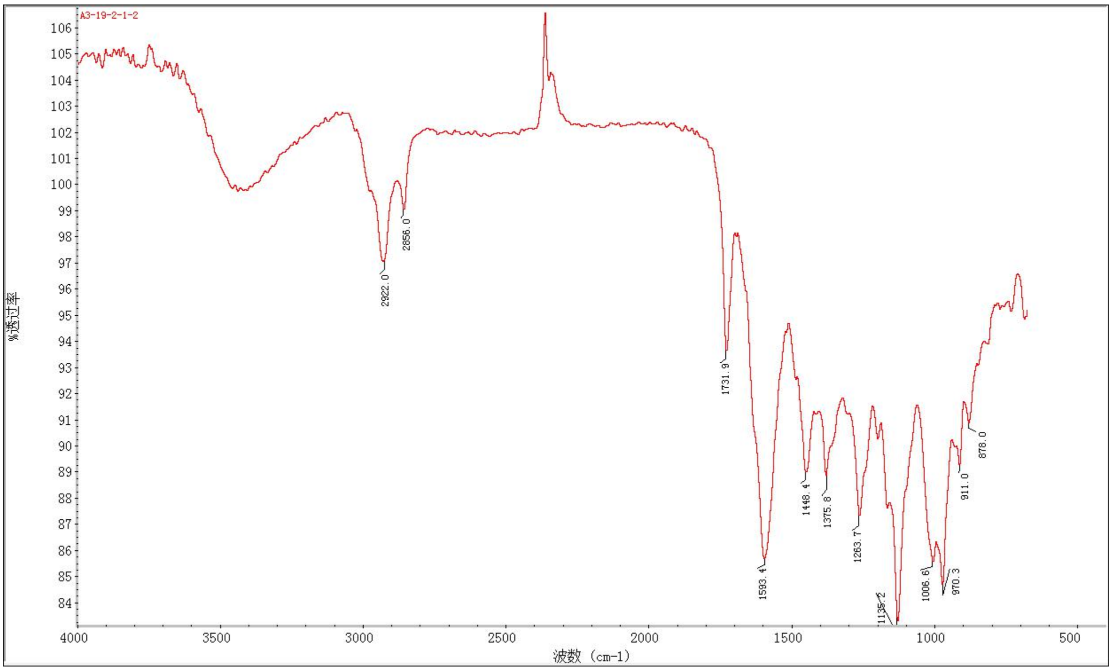

Figure S11 IR spectrum of compound **1**

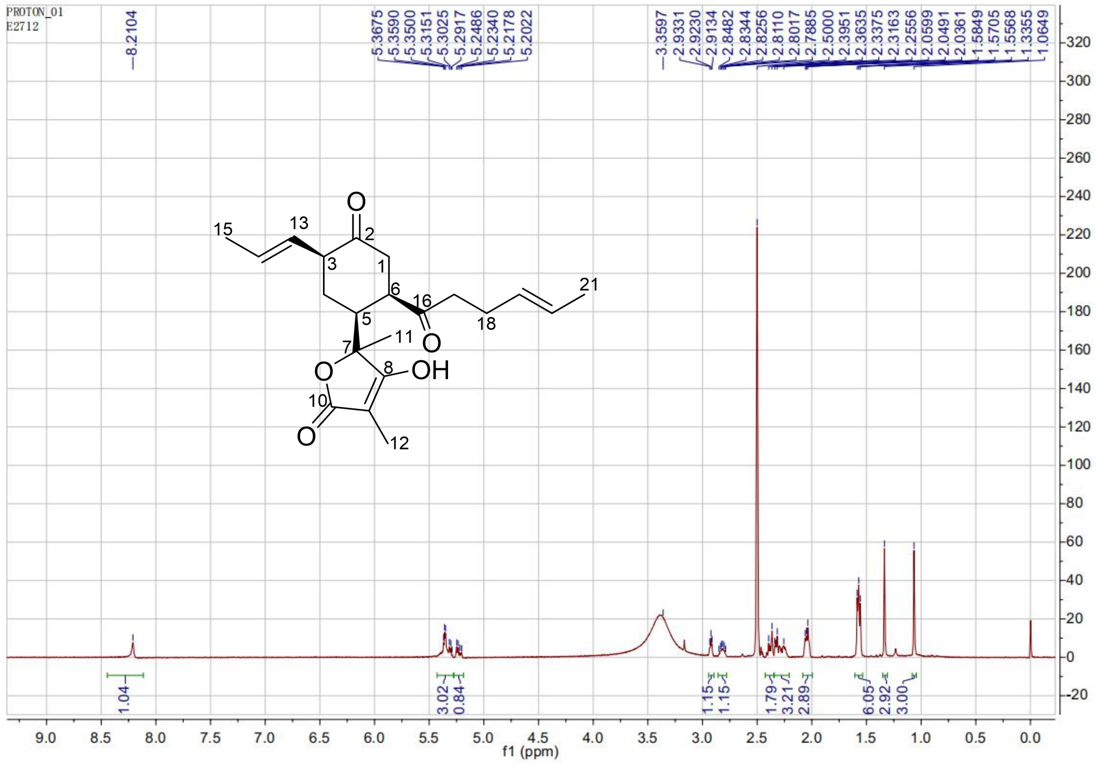

Figure S12 The ^{1}H NMR (500 MHz, DMSO-d_6) spectrum of compound **2**

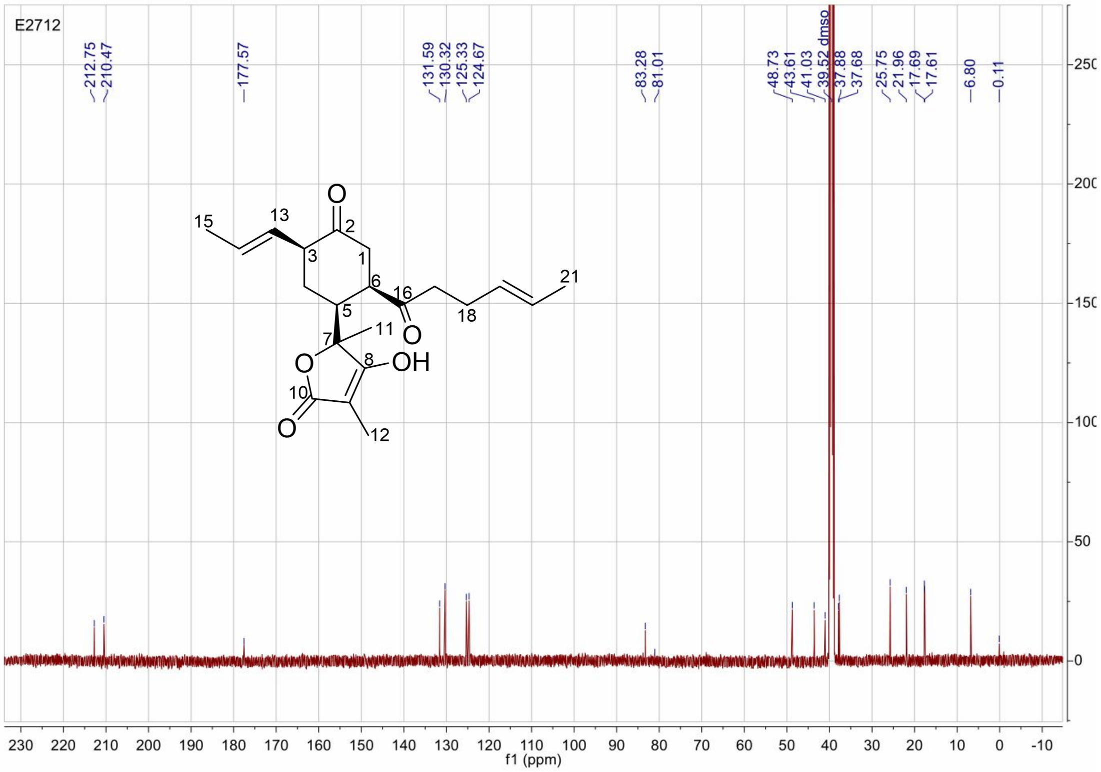

Figure S13 The ^{13}C NMR (125 MHz, DMSO-d_6) spectrum of compound **2**

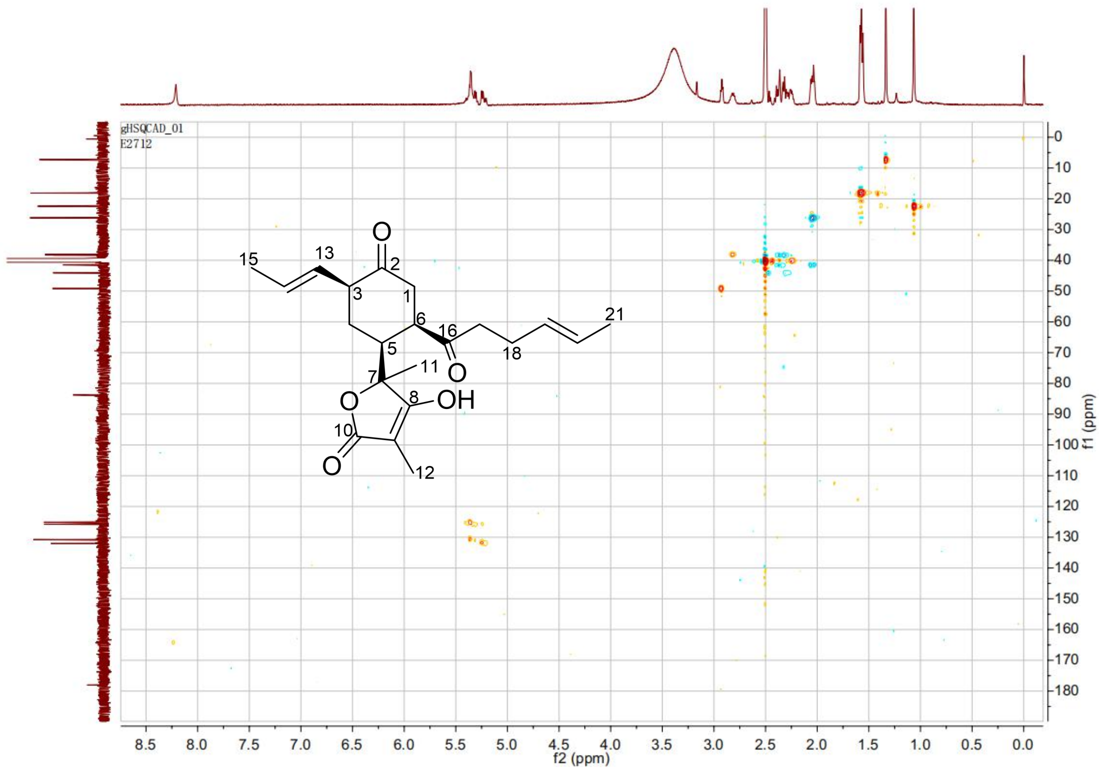

Figure S14 The HSQC (500 MHz, DMSO-d_6) spectrum of compound **2**

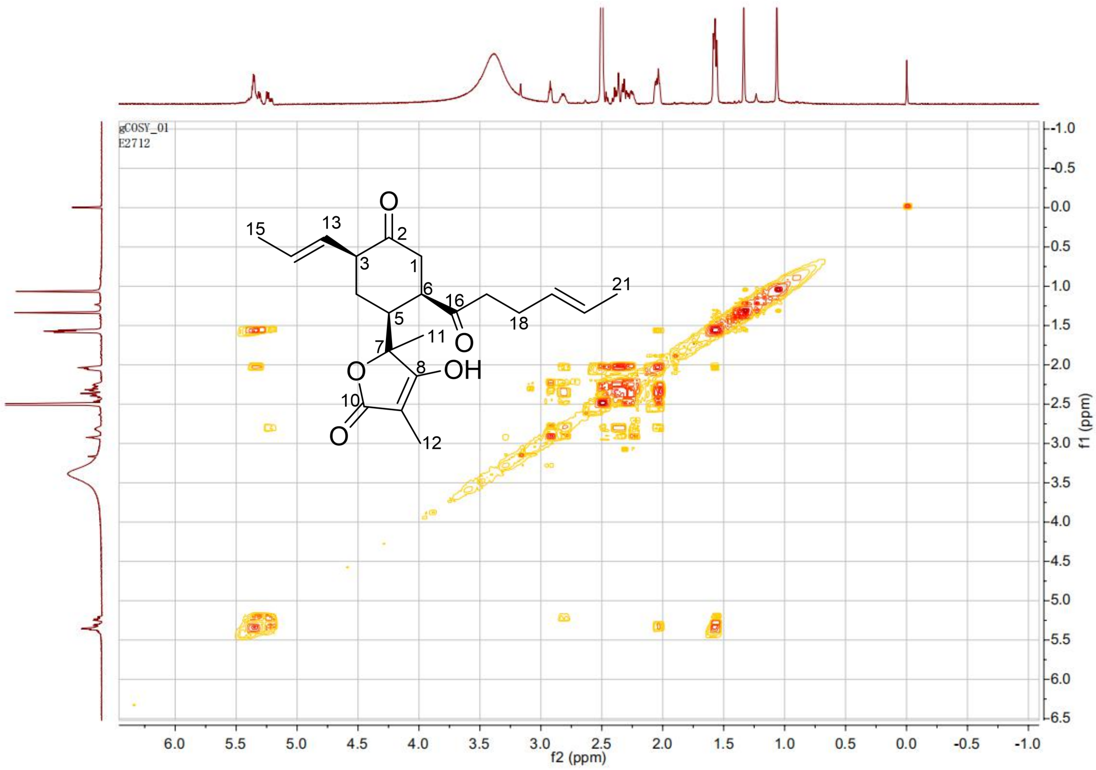

Figure S15 The ^{1}H-^{1}H COSY (500 MHz, DMSO-d_6) spectrum of compound **2**

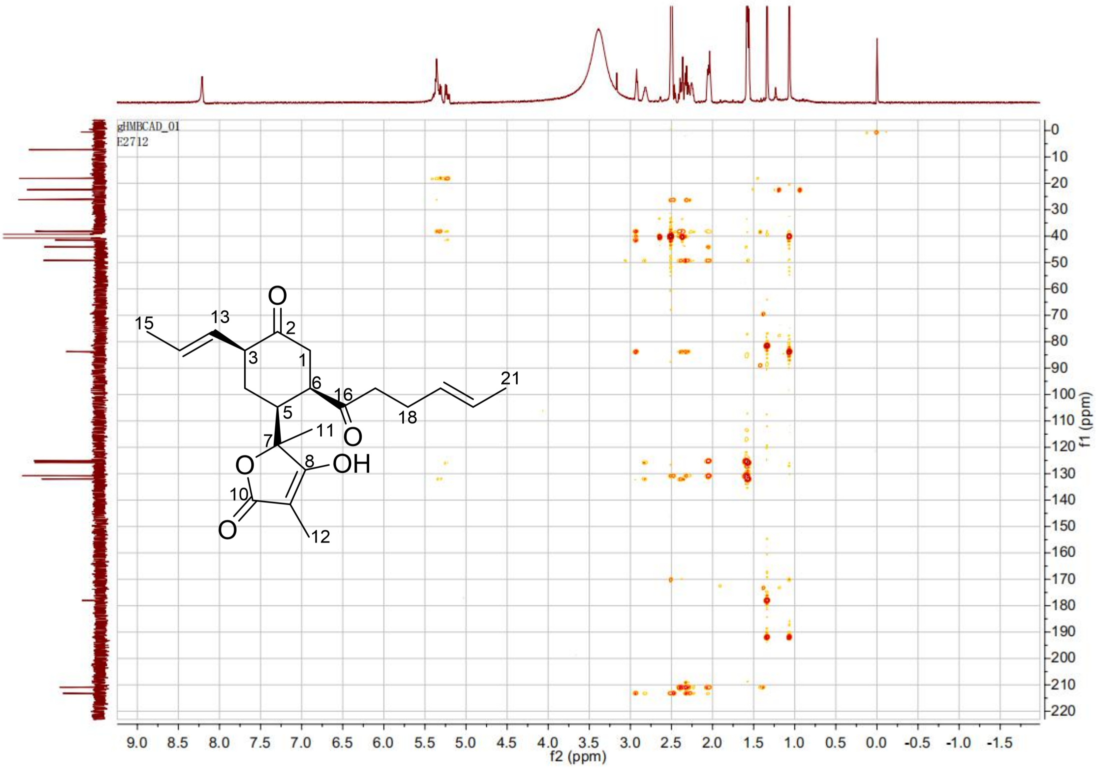

Figure S16 The HMBC (500 MHz, DMSO-d_6) spectrum of compound **2**

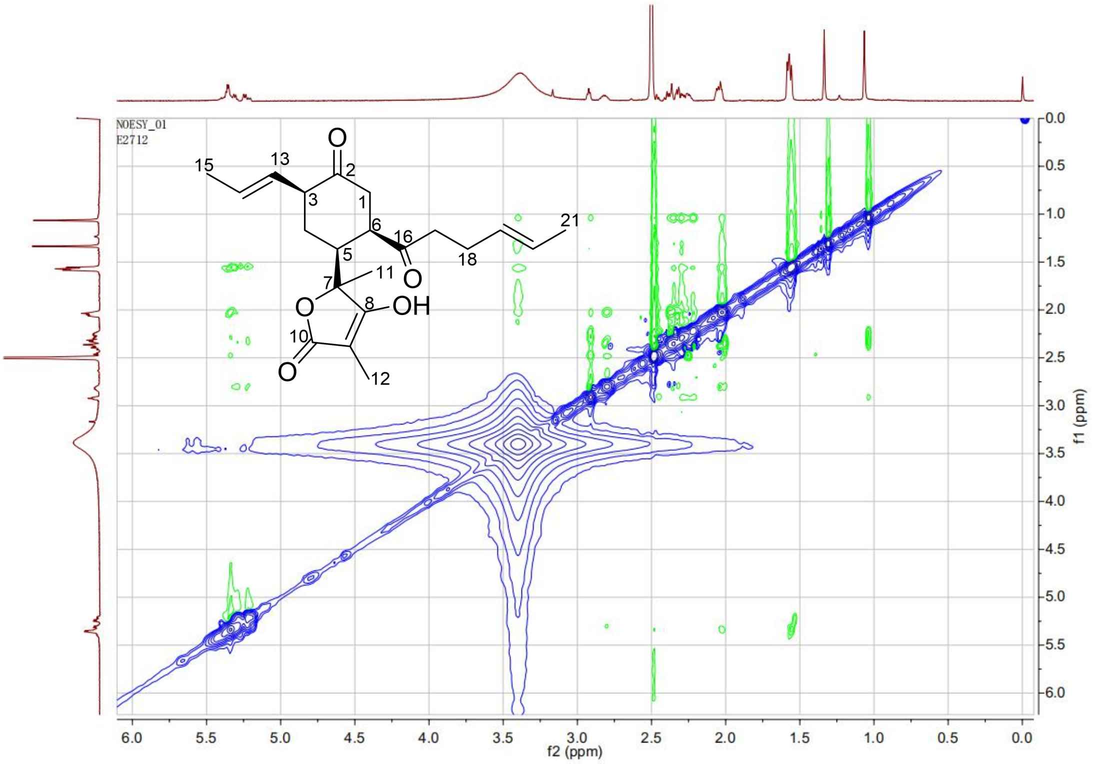

Figure S17 The NOESY (500 MHz, DMSO-d_6) spectrum of compound **2**

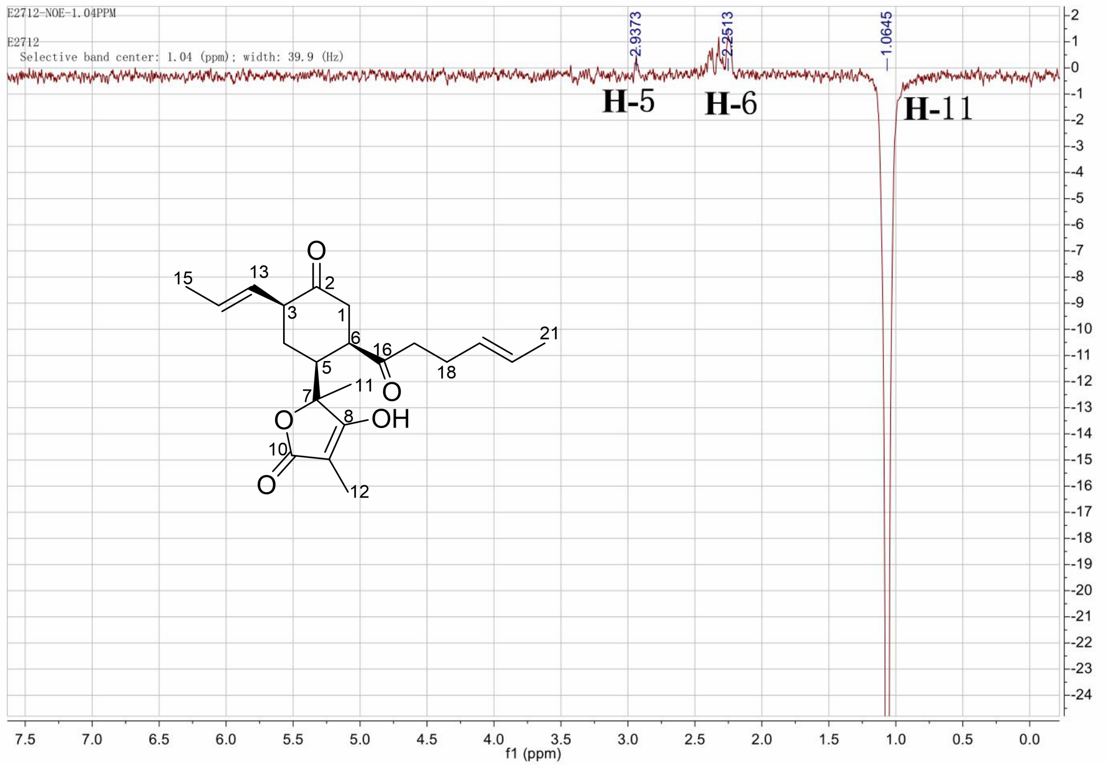

Figure S18 The 1D NOE (500 MHz, DMSO-d_6) spectrum of compound **2** (1)

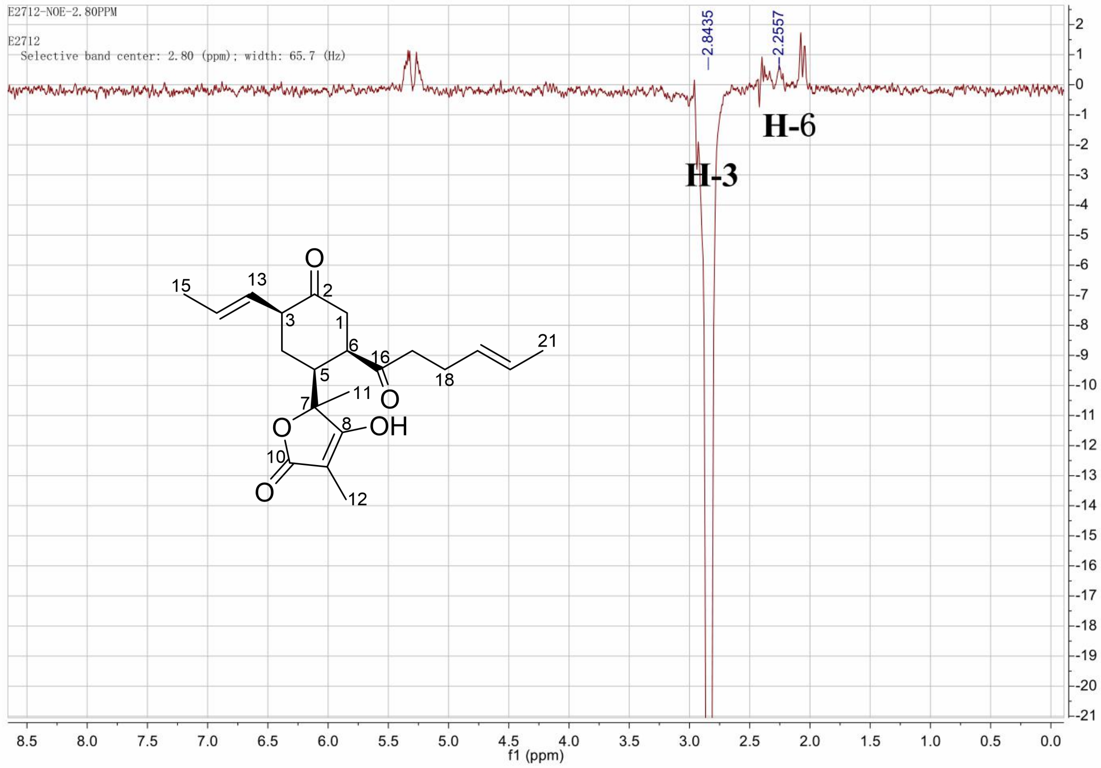

Figure S19 The 1D NOE (500 MHz, DMSO-d_6) spectrum of compound **2** (2)

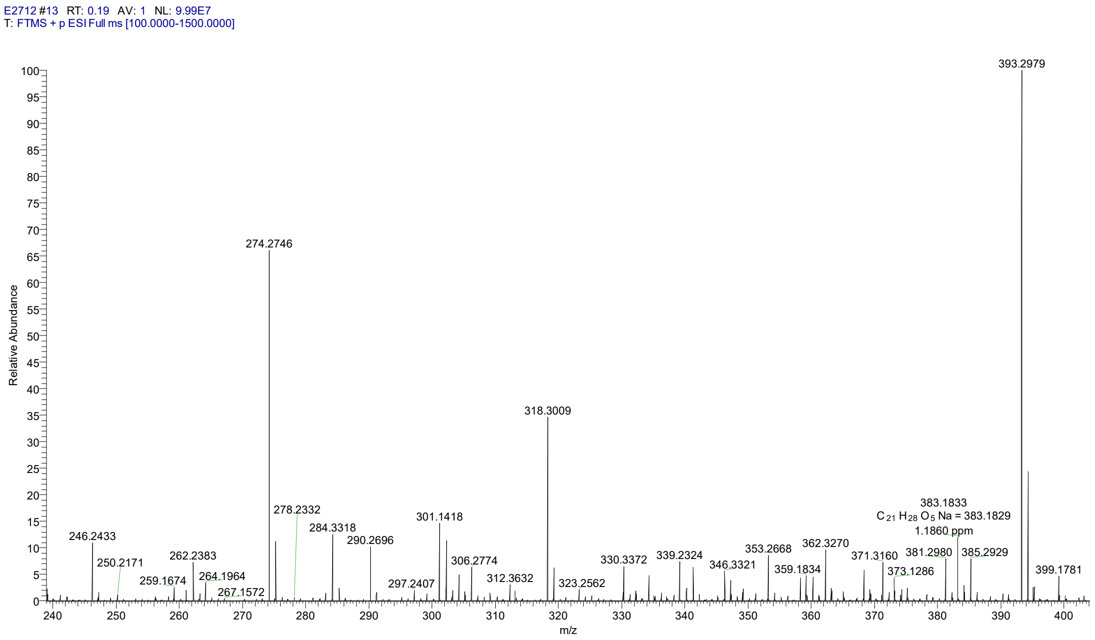

Figure S20 The HRESIMS spectrum of compound **2**

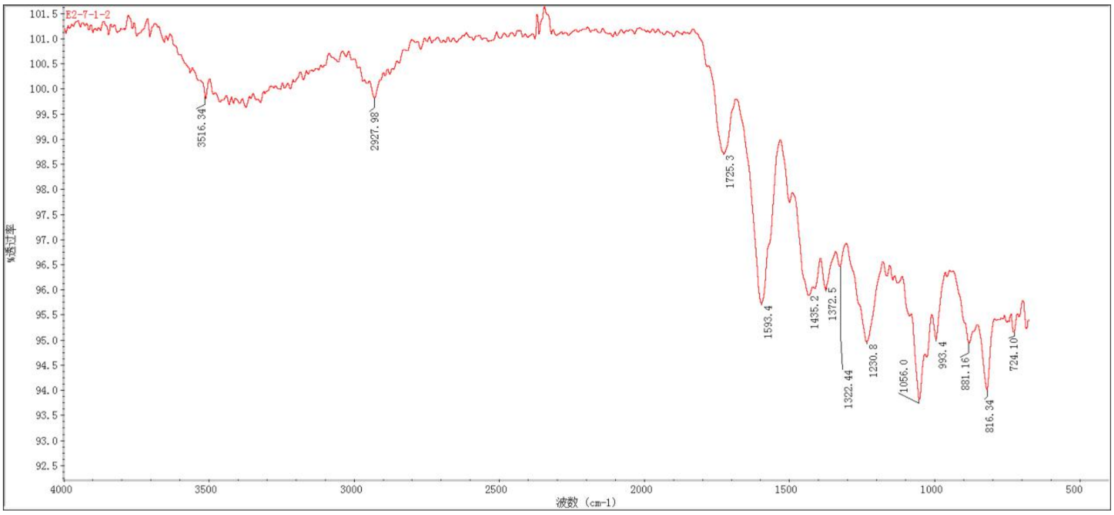

Figure S21. IR spectrum of compound **2**

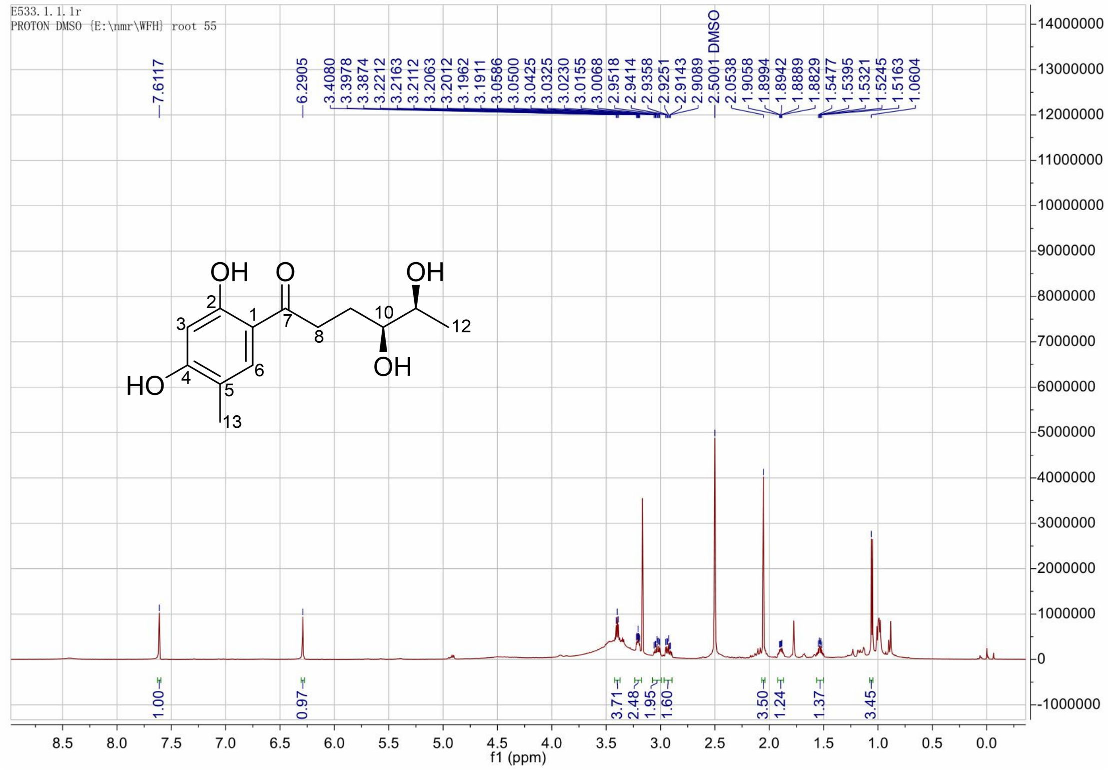

Figure S22 The ^{1}H NMR (600 MHz, DMSO-d_6) spectrum of compound **3**

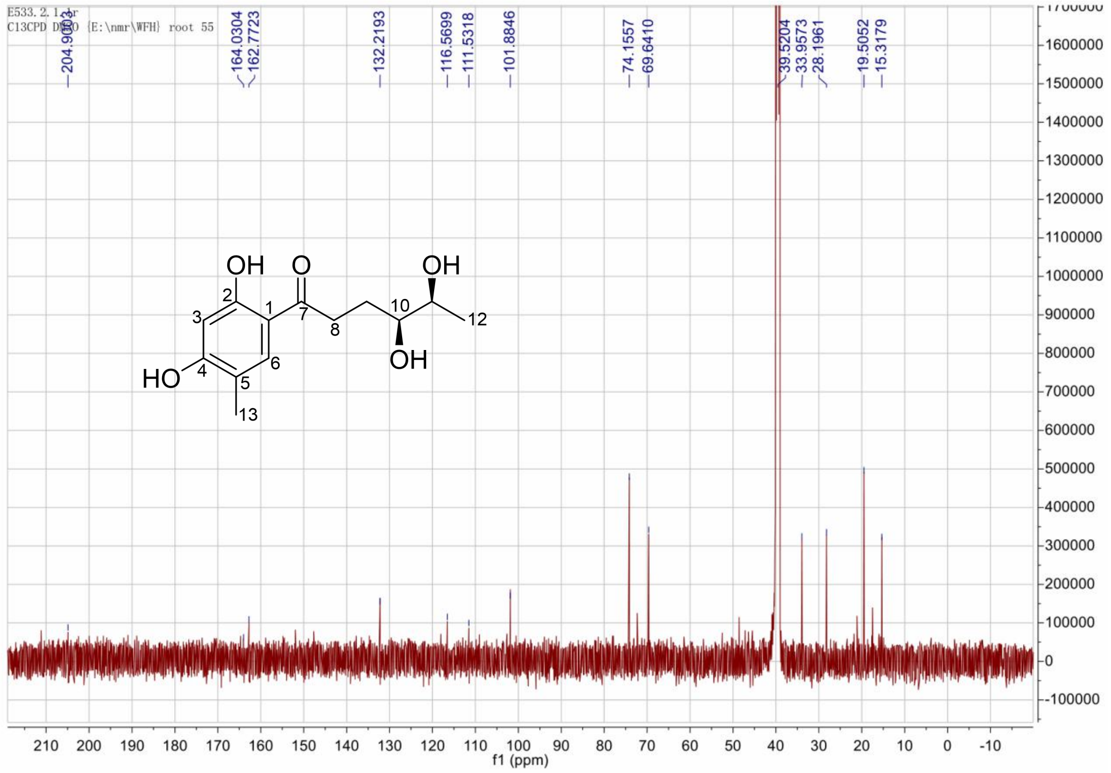

Figure S23 The ^{13}C NMR (150 MHz, DMSO-d_6) spectrum of compound **3**

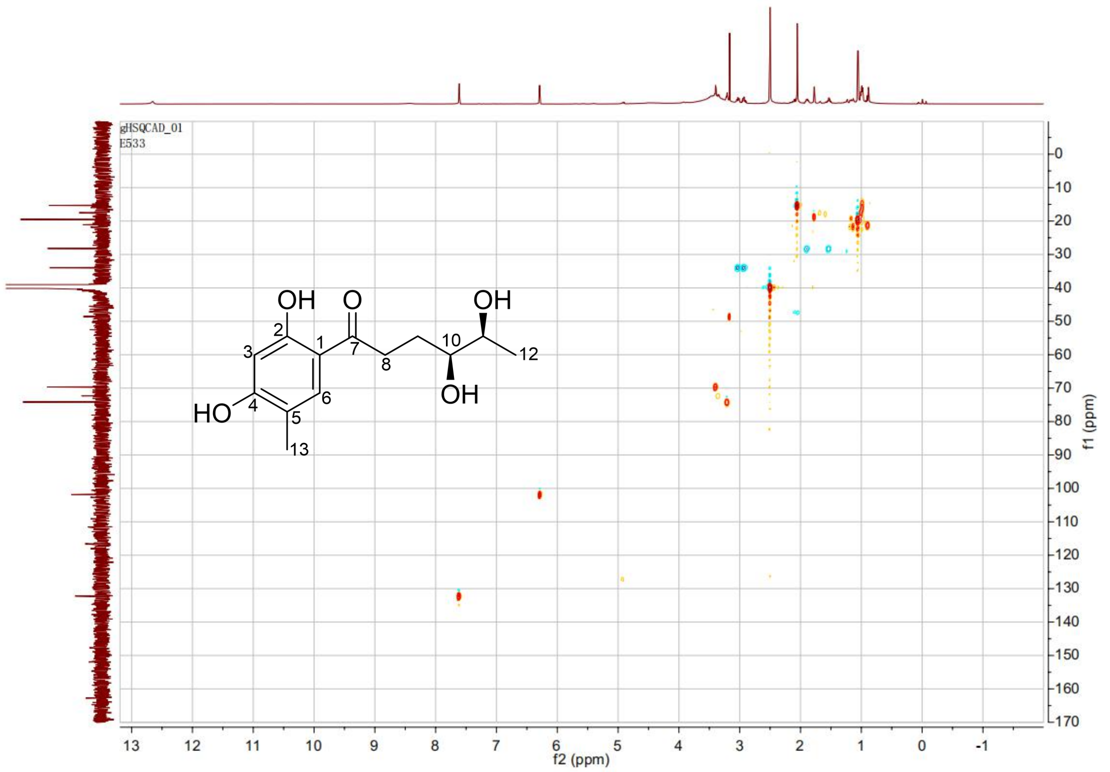

Figure S24 The HSQC (500 MHz, DMSO-d_6) spectrum of compound **3**

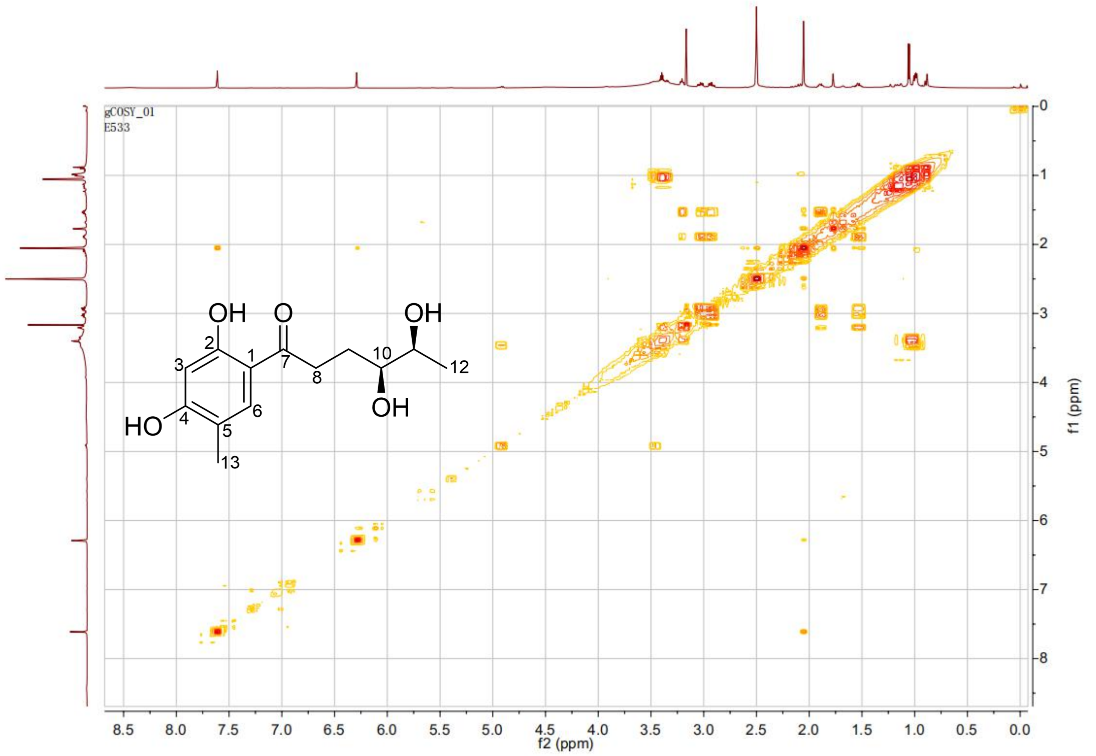

Figure S25 The ^{1}H-^{1}H COSY (500 MHz, DMSO-d_6) spectrum of compound **3**

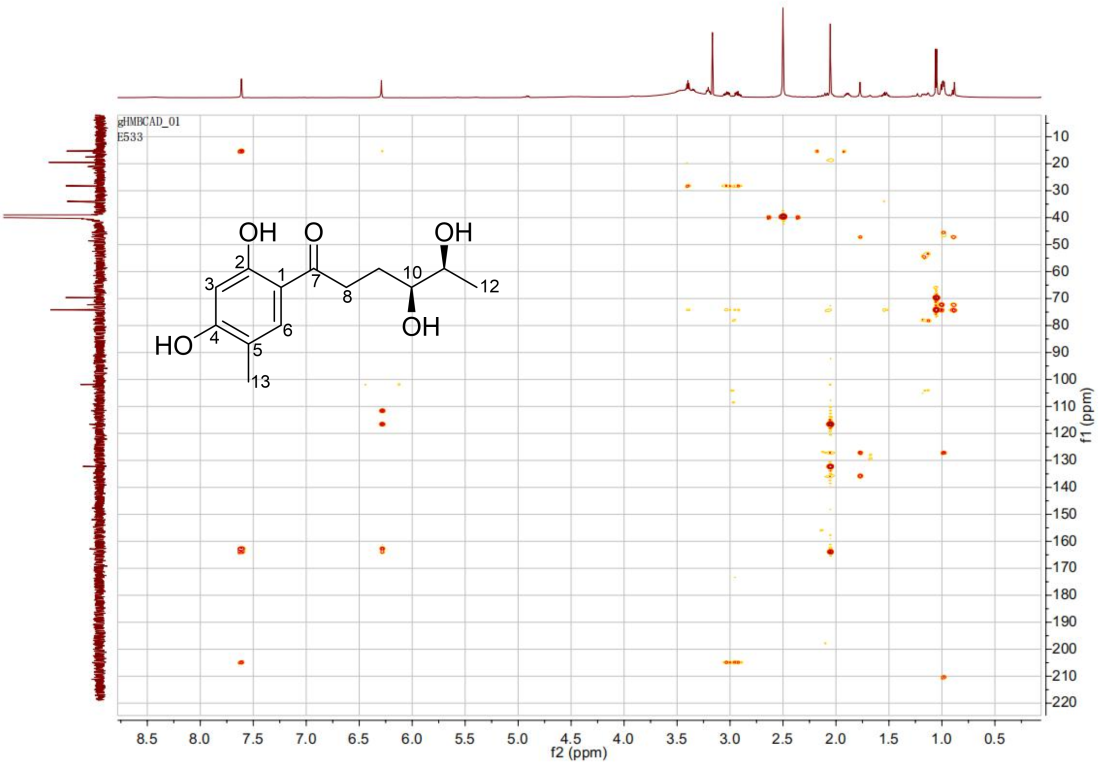

Figure S26 The HMBC (500 MHz, DMSO-d_6) spectrum of compound **3**

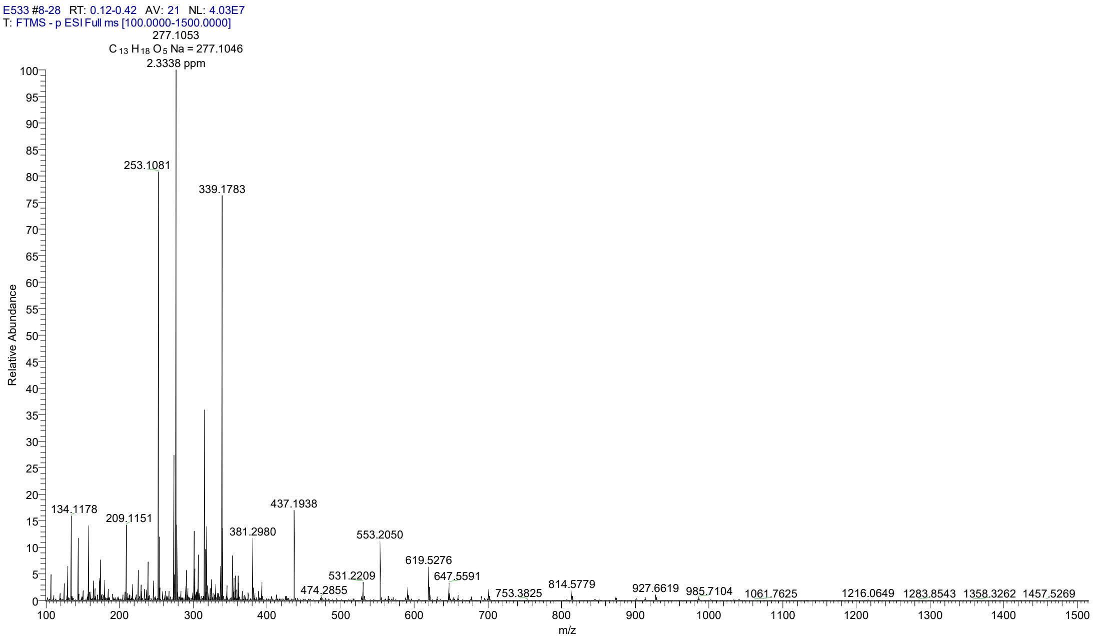

Figure S27 The HRESIMS spectrum of compound **3**

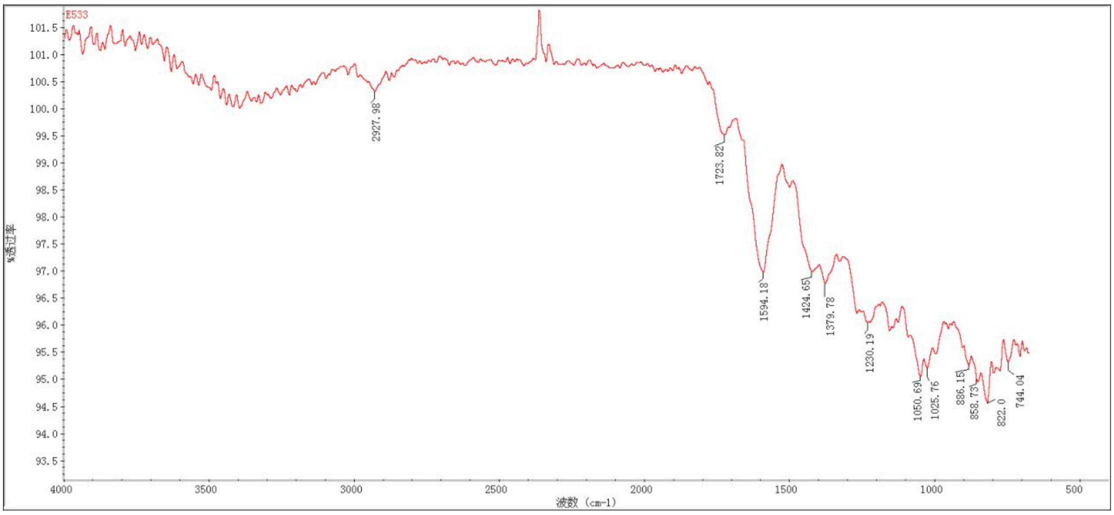

Figure S28 IR spectrum of compound **3**

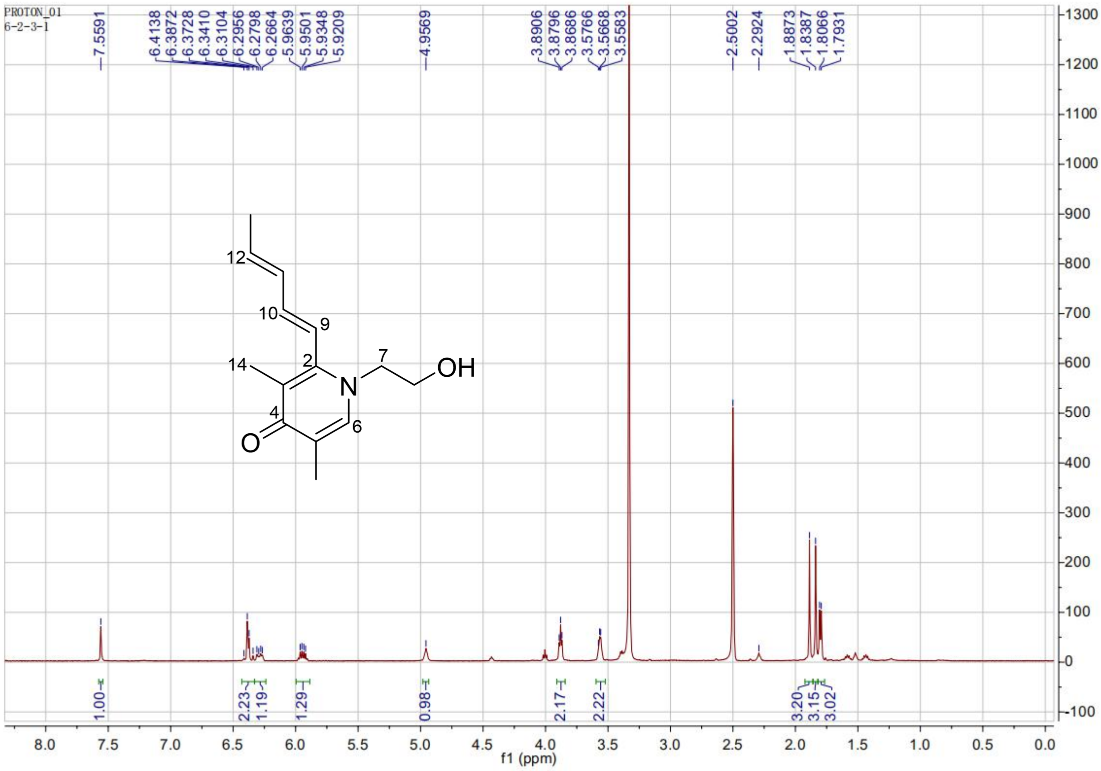

Figure S29 The ^{1}H NMR (500 MHz, DMSO-d_6) spectrum of compound **4**

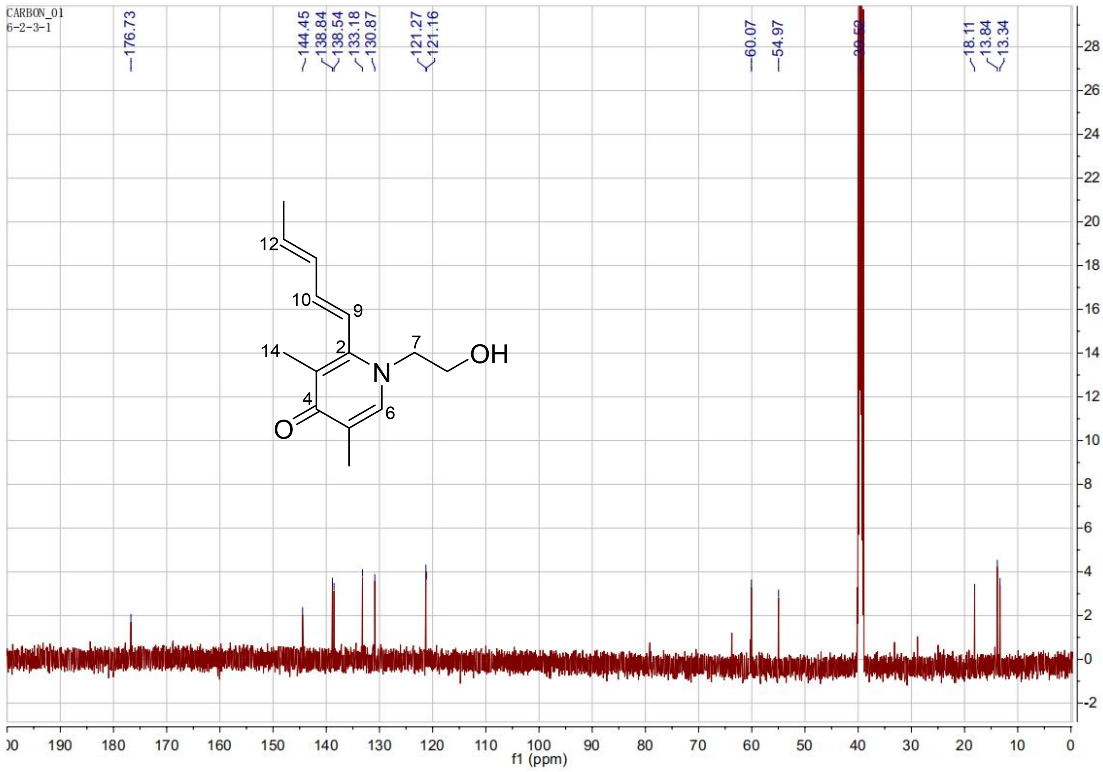

Figure S30 The ^{13}C NMR (125 MHz, DMSO-d_6) spectrum of compound **4**

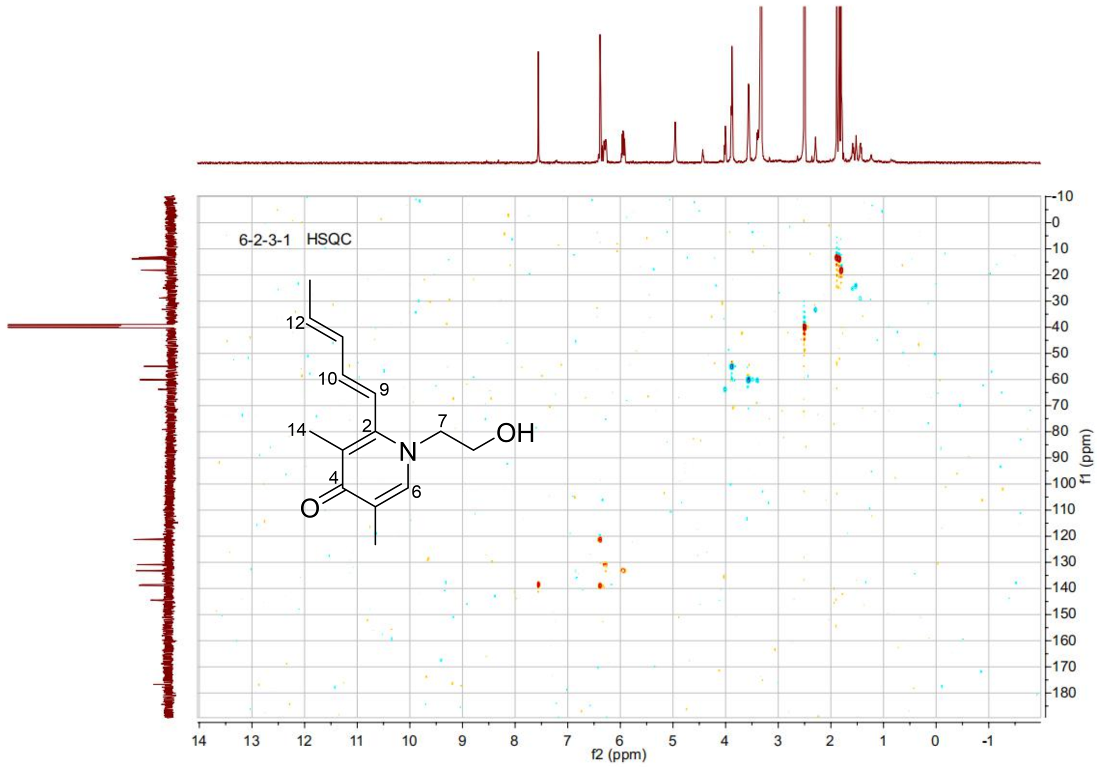

Figure S31 The HSQC (500 MHz, DMSO-d_6) spectrum of compound **4**

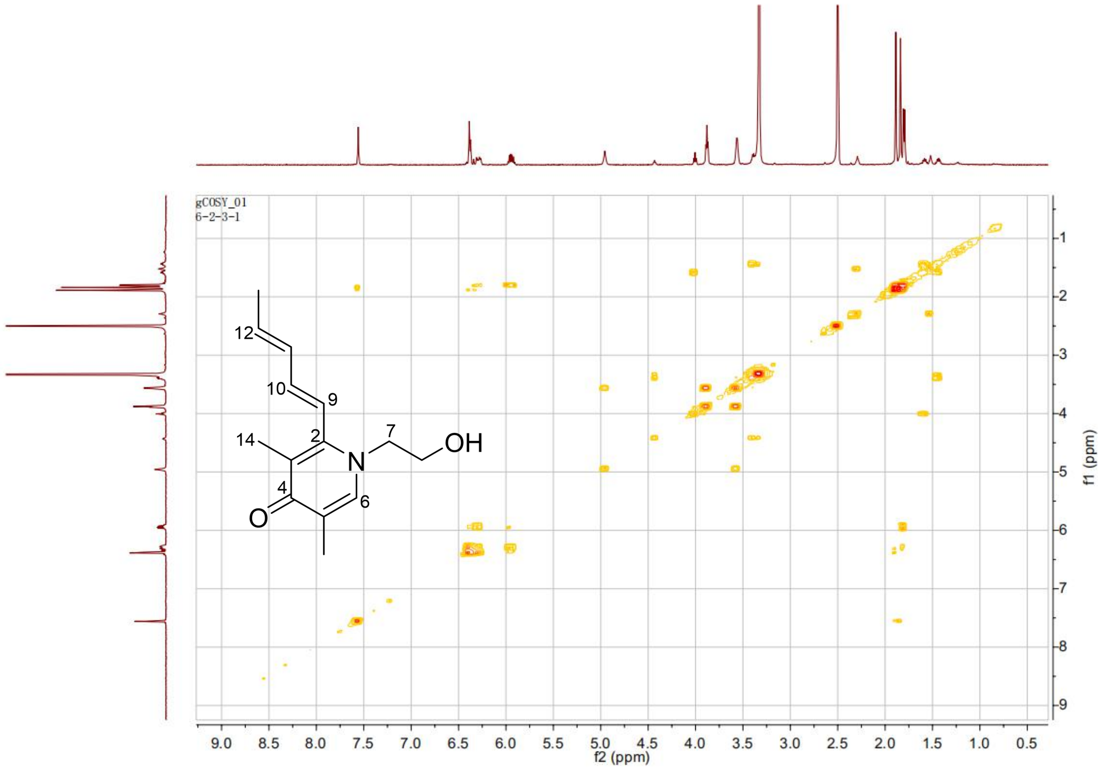

Figure S32 The ^{1}H-^{1}H COSY (500 MHz, DMSO-d_6) spectrum of compound **4**

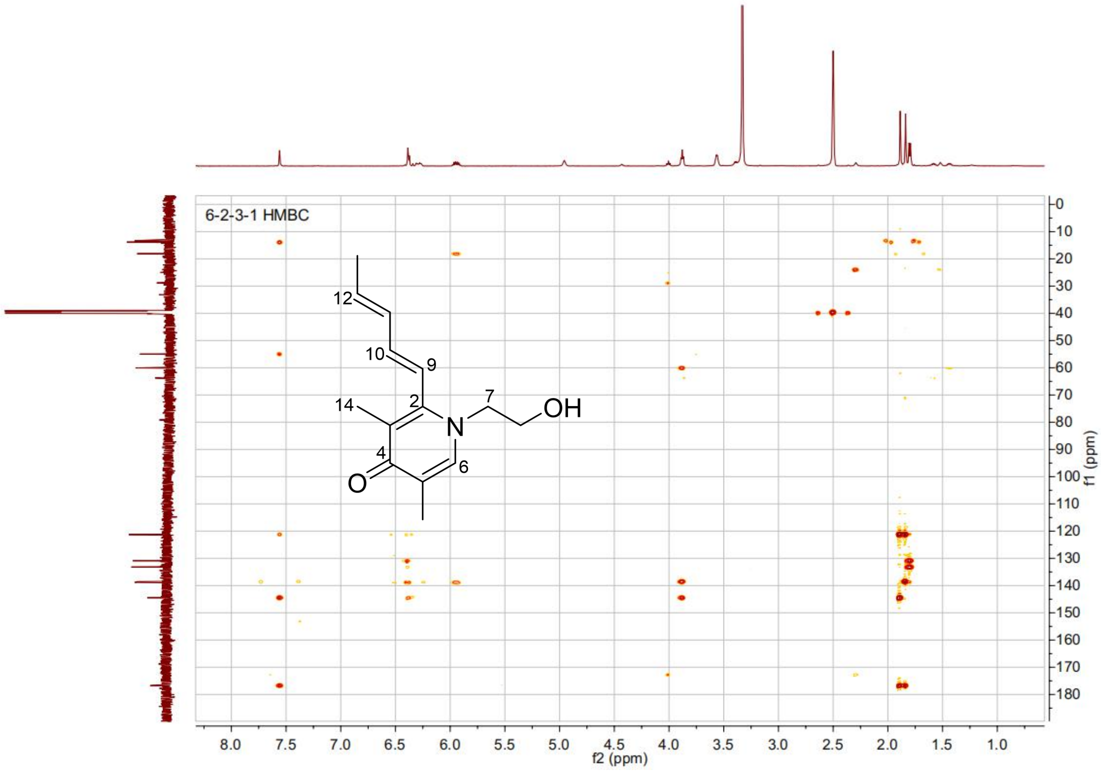

Figure S33 The HMBC (500 MHz, DMSO-d_6) spectrum of compound **4**

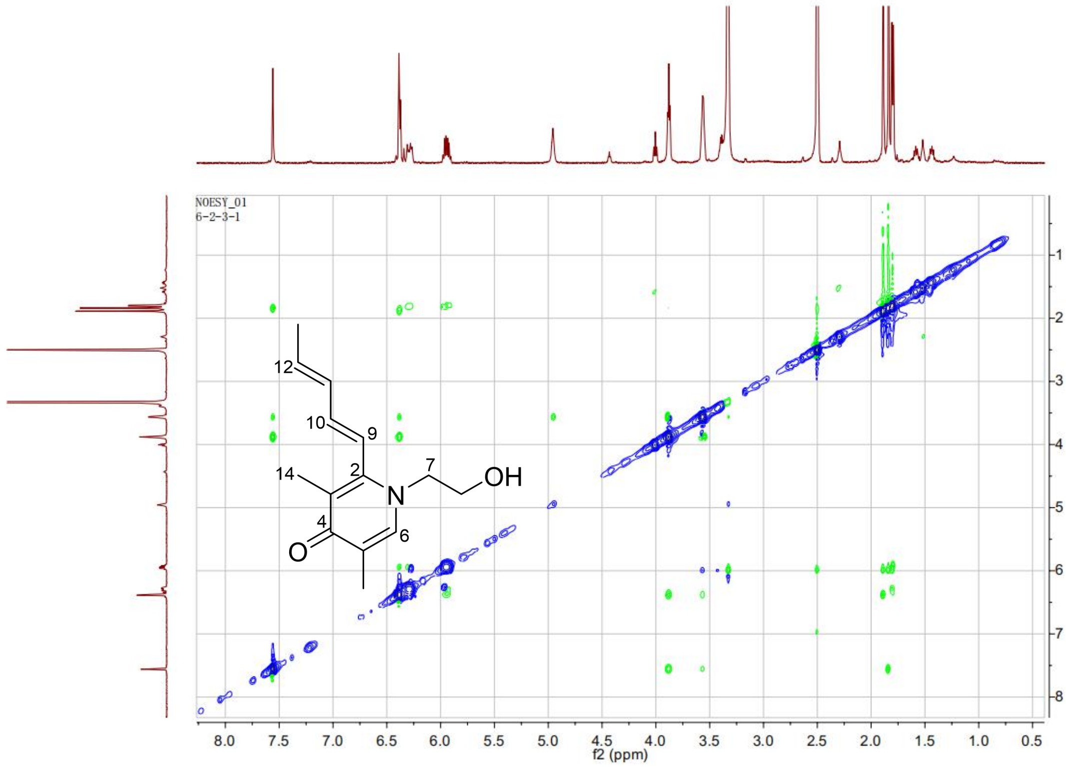

Figure S34 The NOESY (500 MHz, DMSO-d_6) spectrum of compound **4**

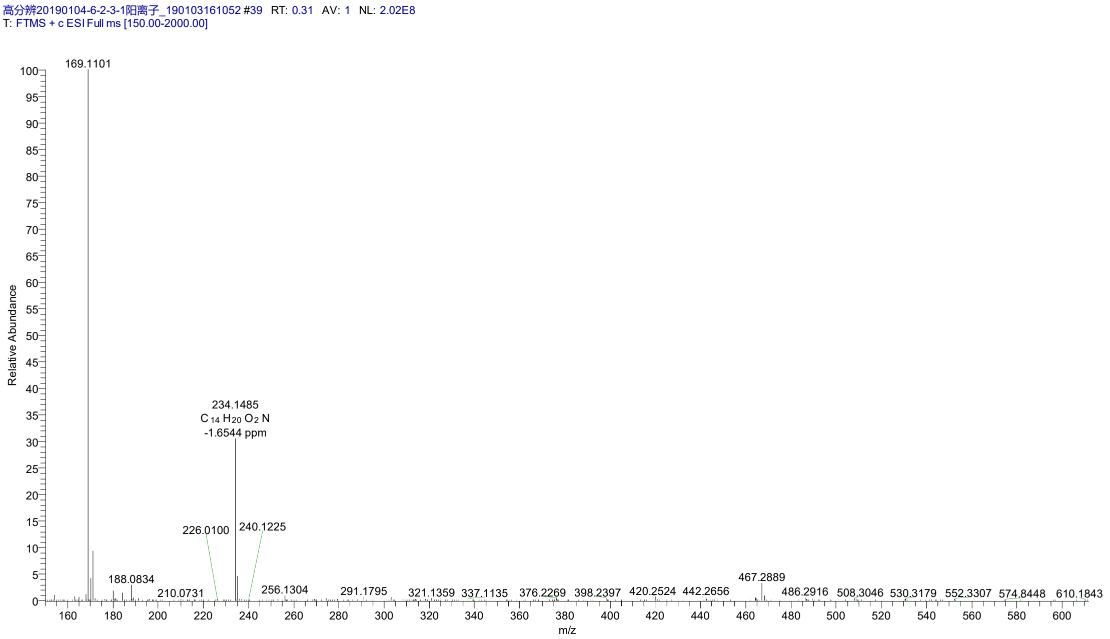

Figure S35 The HRESIMS spectrum of compound **4**

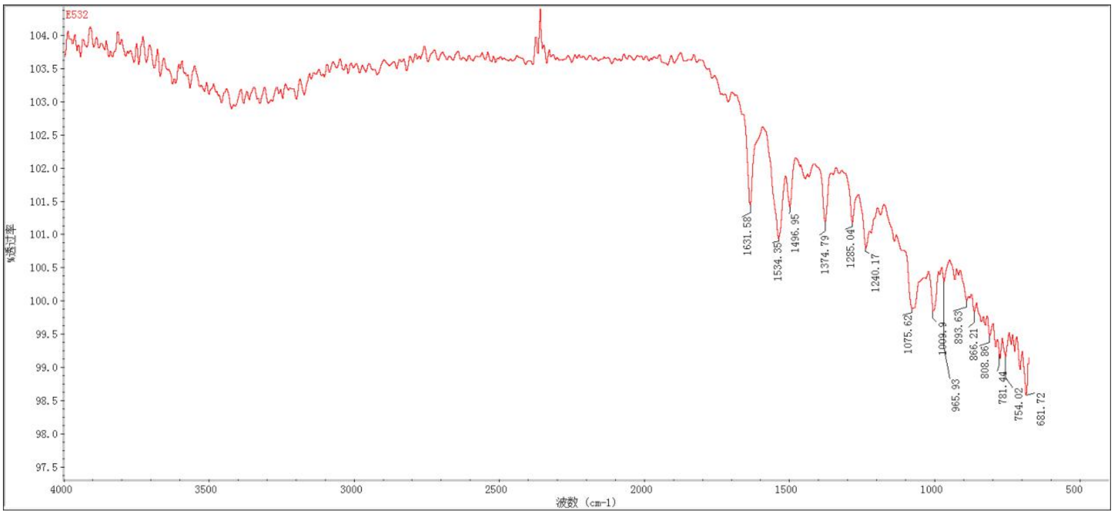

Figure S36 IR spectrum of compound **4**

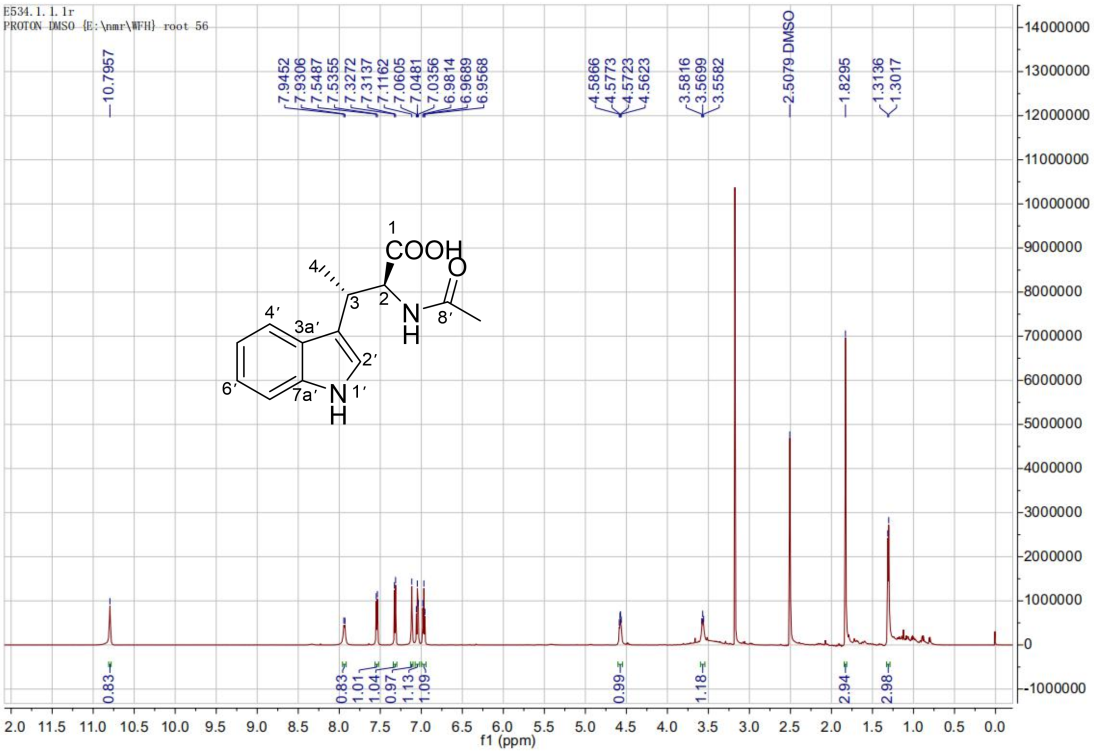

Figure S37 The ^{1}H NMR (600 MHz, DMSO-d_6) spectrum of compound **5**

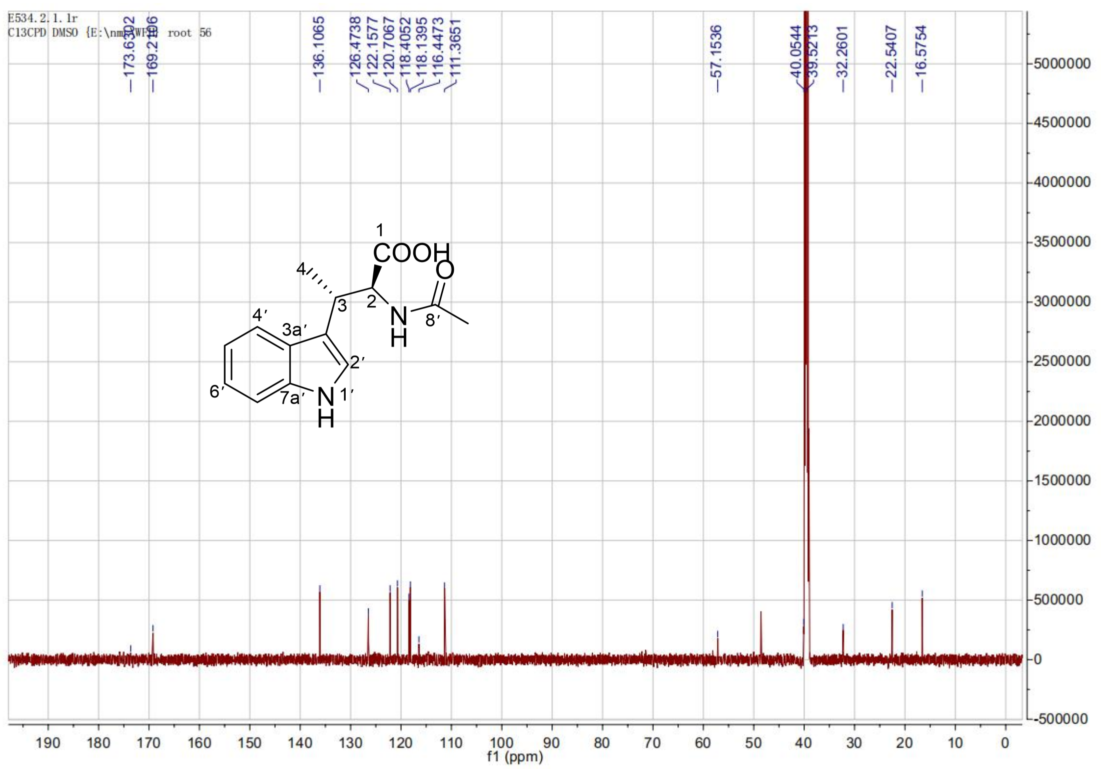

Figure S38 The ^{13}C NMR (150 MHz, DMSO-d_6) spectrum of compound **5**

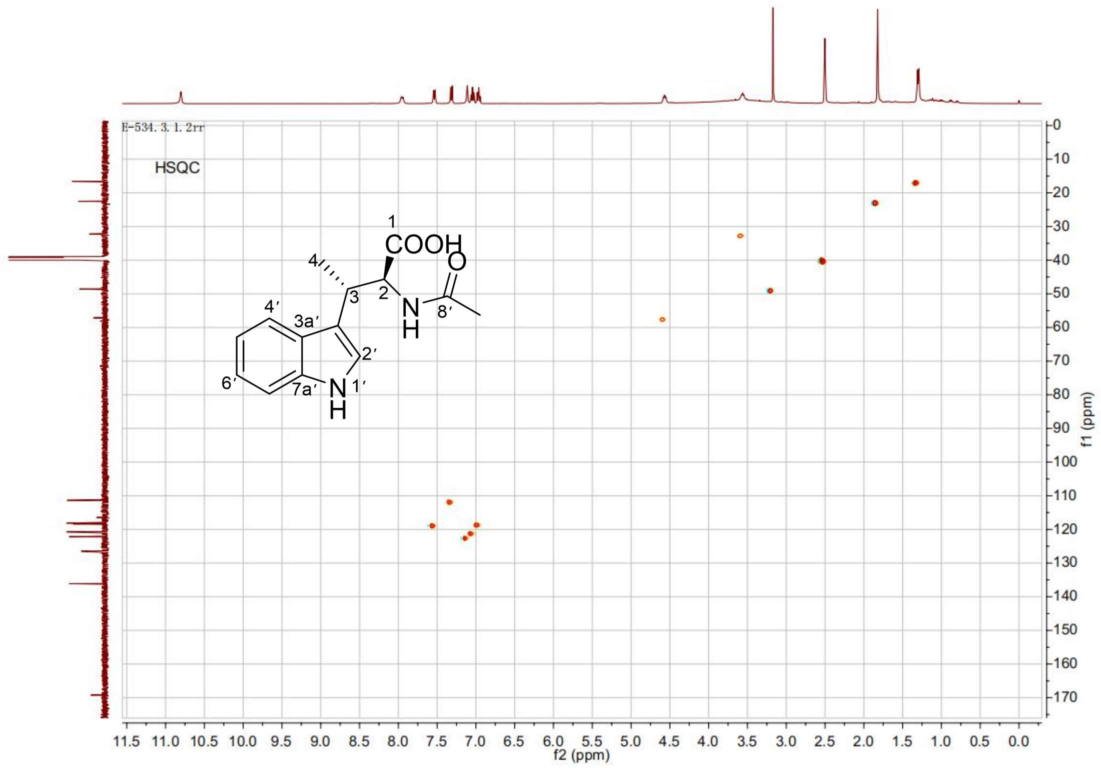

Figure S39 The HSQC (400 MHz, DMSO-d_6) spectrum of compound **5**

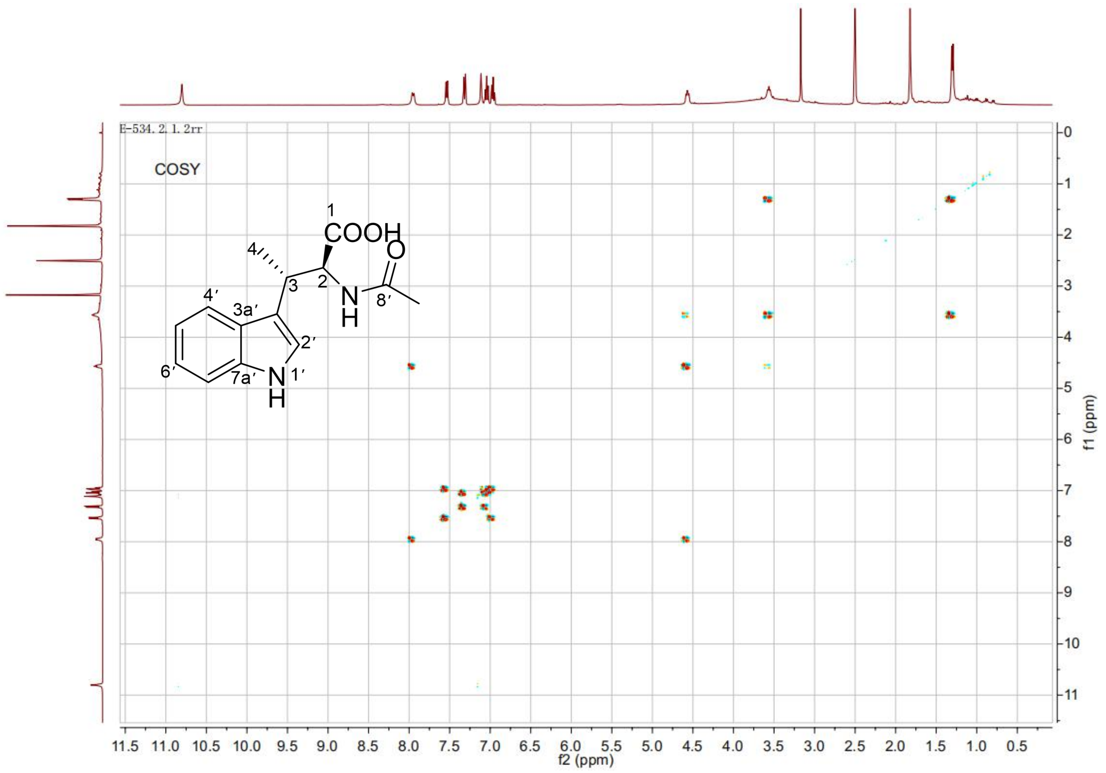

Figure S40 The ^{1}H-^{1}H COSY (400 MHz, DMSO-d_6) spectrum of compound **5**

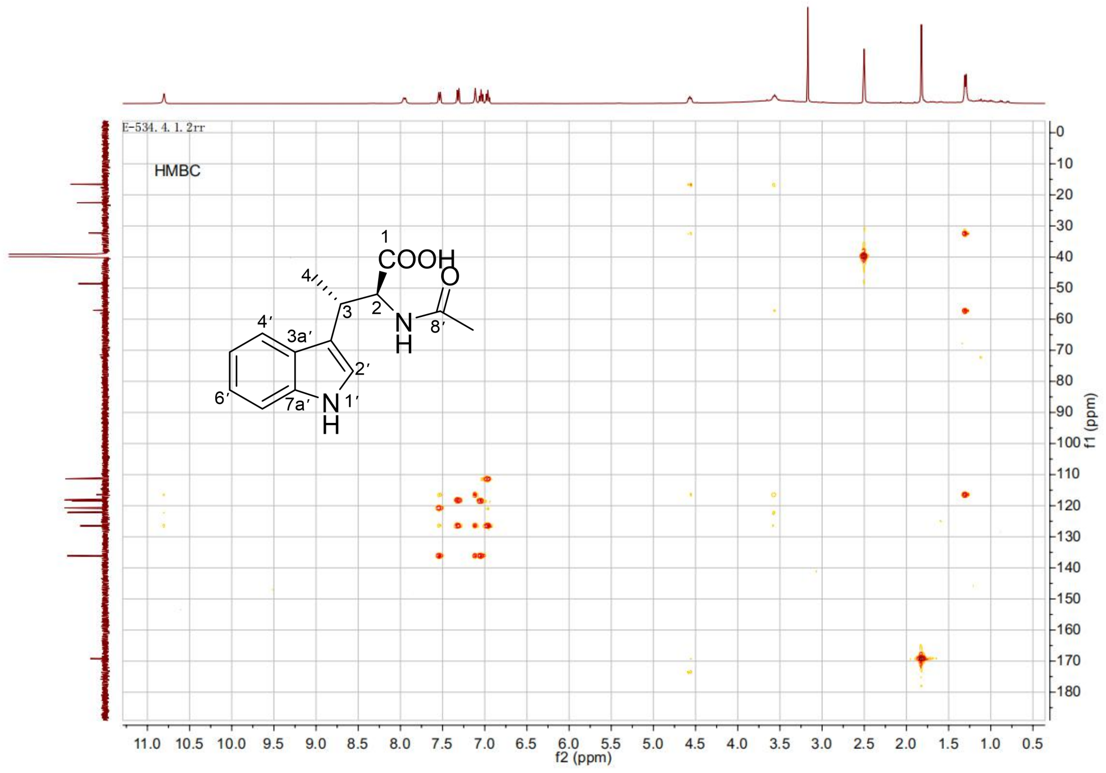

Figure S41 The HMBC (400 MHz, DMSO-d_6) spectrum of compound **5**

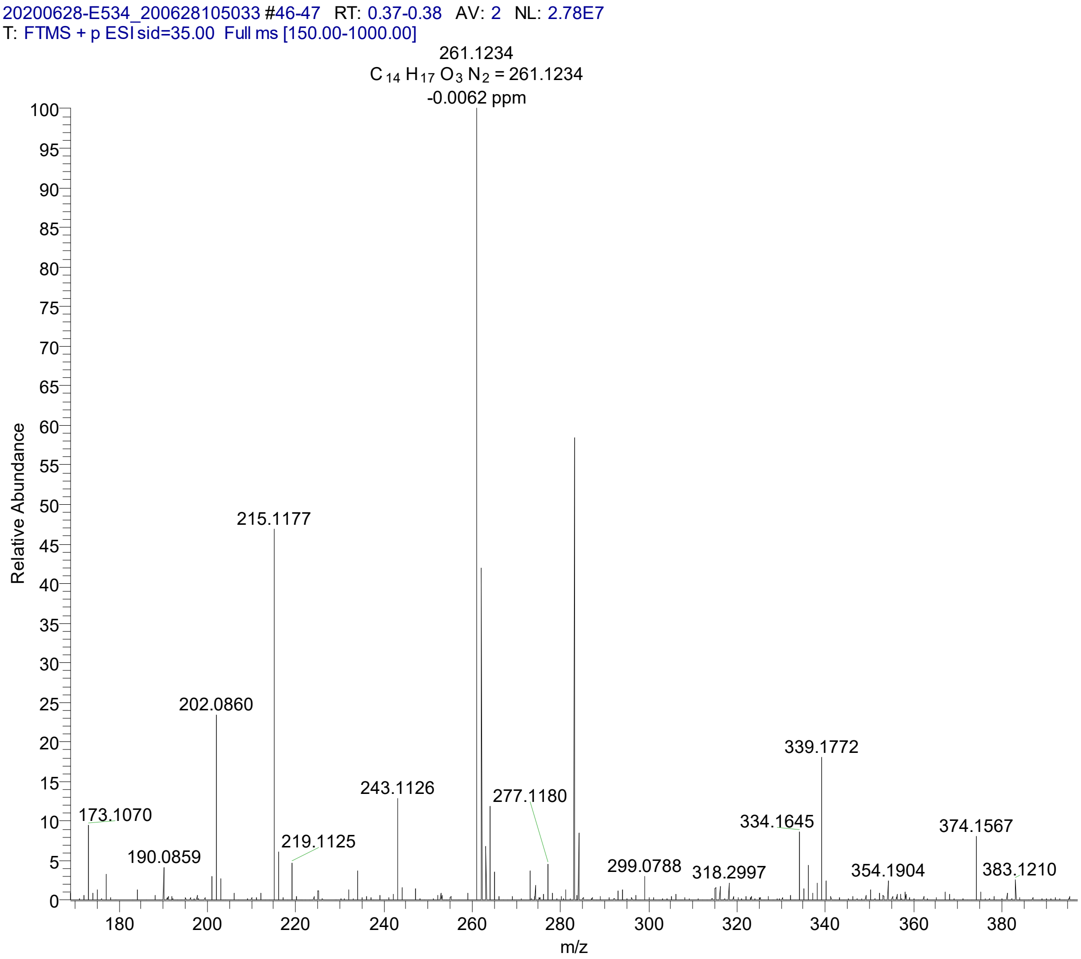

Figure S42 The HRESIMS spectrum of compound **5**

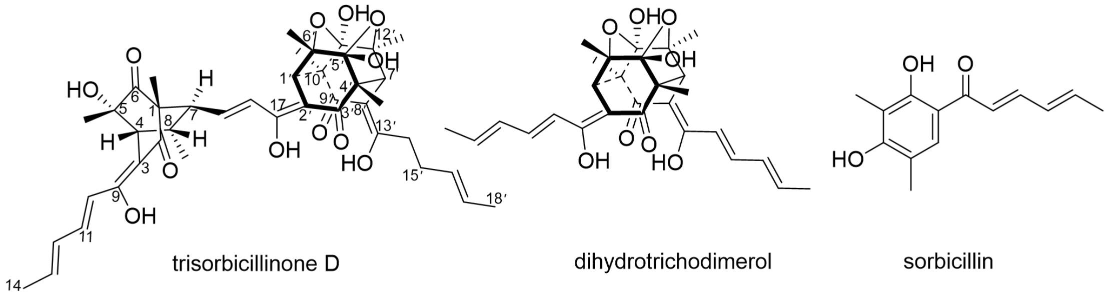

Figure S43 The structures of trisorbicillinone D[S4], dihydrotrichodimerol[S5], and sorbicillin[S6,7]

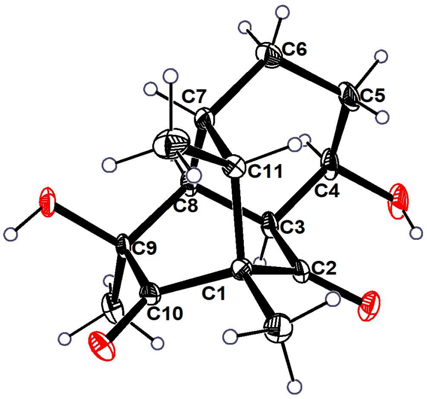

Figure S44. ORTEP diagram for the single-crystal X-ray geometry of **13**

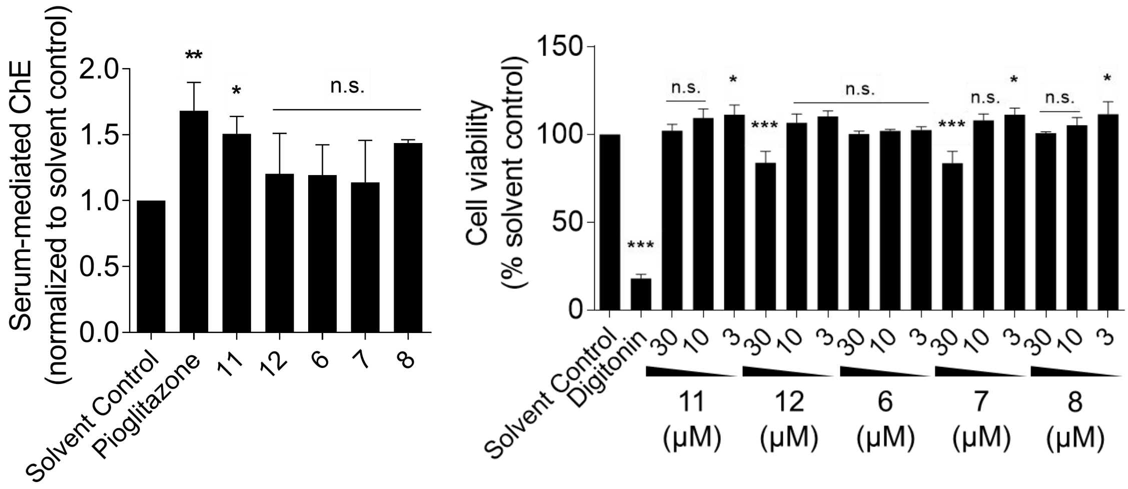

A B

(A) J774A.1 macrophages were labeled with fluorescent BODIPY-cholesterol for 24 h, and treated with the indicated components for another 24 h. After incubation, cells were treated with and without serum for further 6 h to induce cholesterol efflux from macrophages. Pioglitazone (3 μM) was used as positive control. (B) J774A.1 macrophages were treated with the respective compounds at the indicated concentrations for 24 h. After incubation, cells were washed and incubated with 10μg/mL resazurin for further 4 h. Fluorescence derived from the converted resazurin was measured and evaluated as cell viability. Digitonin (at 50μg/mL), was used as positive control. Data are expressed as mean ± SD of three independent experiments and evaluated by one-way ANOVA analysis with the Bonferroni post-test. *$p < 0.05$, **$p < 0.01$ compared with solvent vehicle control (DMSO), n.s. not significant vs. DMSO.

Materials and methods:

Cell Culture

J774A.1 cells were obtained from ATCC and cultured in T175 flasks with phenol red Roswell Park Memorial Institute (RPMI) 1640 medium (Gibco, China) supplemented with 2 mM glutamine, 100 U/mL benzylpenicillin, 100μg/mL streptomycin, and 10% fetal bovine serum (PAN seratech, Germany). Cells were maintained at 37 °C with 5% CO_2 in a humidified atmosphere. When needed, J774A.1 cells were seeded at a density of 0.2×10^6 per mL in 96-well-plates for experiments.

Resazurin Conversion Assay

J774A.1 cells were treated with the respective compounds (**6**, **7**, **8**, **11**, **12**) at concentrations of 3, 10, and 30 μM for 24 h. After incubation, cells were washed with PBS and incubated with resazurin (TOPSCIENCE, China) for another 4 h. Fluorescence (Ex/Em = 535/580 nm) of the generated resorufin was quantified as a measure of cell viability.

Cholesterol Efflux Assay

THP1 macrophages were labeled with BODIPY-cholesterol and treated with the indicated compounds (10 μM), pioglitazone (3 μM), and solvent vehicle control (DMSO) for 24 h. After incubation, cells were treated with the same compounds with and without mouse serum for further 6 h to induce cholesterol efflux from macrophages.

Statistical Analysis

All experiments were performed three times. The data are presented as the mean ± SD. Statistical evaluation was done by one-way analysis of variance (ANOVA) using GraphPad Prism 7 software (GraphPad Software Inc.). Differences between the vehicle control group and experimental condition with a $p < 0.05$ were considered statistically significant.

Figure S45. Influence of compounds **6**, **7**, **8**, **11**, and **12** on serum-mediated cholesterol efflux and on cell viability in J774A.1 macrophages.

References

(S1) Wang, Z. R.; Li, G.; Ji, L. X.; Wang, H. H.; Gao, H.; Peng, X. P.; Lou, H. X. *Steroids* **2019**, 145, 1−4.

(S2) Miteva, M. A.; Guyon, F.; Tuffery, P. *Nucleic Acids Res.* **2010**, 38, W622−627.

(S3) Stephens, P. J.; Harada, N. *Chirality* **2010**, 22, 229−233.

(S4) Li, D.; Cai, S.; Zhu, T.; Wang, F.; Xiao, X.; Gu, Q. *Tetrahedron* **2010**, 66, 5101−5106.

(S5) Liu, W.; Gu, Q.; Zhu, W.; Cui, C.; Fan, G. *J. Antibiot.* **2005**, 58, 621−624.

(S6) Cram, D. J. *J. Am. Chem. Soc*. **1948**, 70, 4240−4243.

(S7) Cram, D. J.; Tishler, M. *J. Am. Chem. Soc.* **1948**, 70, 4238−4239.
